# Interannual variations in needle and sapwood traits of *Pinus edulis* branches under an experimental drought

**DOI:** 10.1002/ece3.3743

**Published:** 2018-01-05

**Authors:** Marceau Guérin, Dario Martin‐Benito, Georg von Arx, Laia Andreu‐Hayles, Kevin L. Griffin, Rayann Hamdan, Nate G. McDowell, Robert Muscarella, William Pockman, Pierre Gentine

**Affiliations:** ^1^ Department of Earth and Environmental Engineering Columbia University New York NY USA; ^2^ Forest Ecology Department of Environmental Sciences Swiss Federal Institute of Technology ETH Zurich Zürich Switzerland; ^3^ Forest Research Center (INIA‐CIFOR) Madrid Spain; ^4^ Tree‐ring Laboratory Lamont‐Doherty Earth Observatory of Columbia University Palisades NY USA; ^5^ Swiss Federal Institute for Forest, Snow and Landscape Research WSL Birmensdorf Switzerland; ^6^ Climatic Change and Climate Impacts Institute for Environmental Sciences Geneva Switzerland; ^7^ Department of Earth and Environmental Sciences Lamont‐Doherty Earth Observatory of Columbia University Palisades NY USA; ^8^ Ecole Polytechnique Palaiseau France; ^9^ Atmospheric Sciences and Global Change Division Pacific Northwest National Laboratory Richland WA USA; ^10^ Ecoinformatics & Biodiversity Department of Bioscience Aarhus University Aarhus Denmark; ^11^ Department of Biology University of New Mexico Albuquerque NM USA; ^12^ Department of Earth and Environmental Engineering Earth Institute Columbia University New York NY USA

**Keywords:** functional ratio, Huber value, isohydricity, leaf area, stomatal conductance, xylem

## Abstract

In the southwestern USA, recent large‐scale die‐offs of conifers raise the question of their resilience and mortality under droughts. To date, little is known about the interannual structural response to droughts. We hypothesized that piñon pines (*Pinus edulis*) respond to drought by reducing the drop of leaf water potential in branches from year to year through needle morphological adjustments. We tested our hypothesis using a 7‐year experiment in central New Mexico with three watering treatments (irrigated, normal, and rain exclusion). We analyzed how variation in “evaporative structure” (needle length, stomatal diameter, stomatal density, stomatal conductance) responded to watering treatment and interannual climate variability. We further analyzed annual functional adjustments by comparing yearly addition of needle area (LA) with yearly addition of sapwood area (SA) and distance to tip (*d*), defining the yearly ratios SA:LA and SA:LA/*d*. Needle length (*l*) increased with increasing winter and monsoon water supply, and showed more interannual variability when the soil was drier. Stomatal density increased with dryness, while stomatal diameter was reduced. As a result, anatomical maximal stomatal conductance was relatively invariant across treatments. SA:LA and SA:LA/*d* showed significant differences across treatments and contrary to our expectation were lower with reduced water input. Within average precipitation ranges, the response of these ratios to soil moisture was similar across treatments. However, when extreme soil drought was combined with high VPD, needle length, SA:LA and SA:LA/*d* became highly nonlinear, emphasizing the existence of a response threshold of combined high VPD and dry soil conditions. In new branch tissues, the response of annual functional ratios to water stress was immediate (same year) and does not attempt to reduce the drop of water potential. We suggest that unfavorable evaporative structural response to drought is compensated by dynamic stomatal control to maximize photosynthesis rates.

## 1 INTRODUCTION

In recent years, widespread forest mortality in response to drought has been documented worldwide (Allen, Breshears, & McDowell, 2015). An example of widespread and rapid increase in drought‐induced mortality, or die‐off, was observed for *Pinus edulis* Engelm. across the southwestern USA in response to several years of reduced rainfall and high vapor pressure deficits (VPD) (Allen et al., 2010; Breshears et al., 2009; Williams et al., 2013). Although stomatal closure under drought has been hypothesized to increase mortality through carbon starvation (Breshears et al., 2009; McDowell et al., 2008), more evidences exist for mortality being caused by hydraulic failure (Garcia‐Forner et al., 2016; McDowell et al., 2013; Plaut et al., 2012; Sevanto, McDowell, Dickman, Pangle, & Pockman, 2014). Regardless of the mechanism of drought‐induced decline, maintaining a positive supply of water to the foliage is critical for tree functioning and survival.

Species differ in their carbon allocation strategies resulting from, among other processes, the interaction of leaf phenology, use of stored carbohydrates, and intra‐annual xylem growth (Michelot, Simard, Rathgeber, Dufrêne, & Damesin, 2012) or root growth (Gálvez, Landhäusser, & Tyree, 2011). The diversity in carbon allocation strategies/patterns is expected to result in various growth of leaf vs. sapwood. Furthermore, species differ in the onset and timing of growth of different structural components (shoot, xylem, leaves). The growth of the components can be synchronous or asynchronous (Rossi, Rathgeber, & Deslauriers, 2009) and thus dynamically influences both evaporative surface area and water supply to the leaves. To date, little is documented on how drought types (e.g., soil or atmospheric drought, seasonality, intensity) affect interannual carbon allocation patterns, and only one experimental study was found investigating the response to different drought types across xylem and leaf components (Grossiord et al., 2017). For piñons, drought can impair their structural development through a reduction in xylem development (Hartmann, Ziegler, Kolle, & Trumbore, 2013), needles and shoots length (Adams et al., 2015; Grossiord et al., 2016), and the number of needles (Clifford, Royer, Cobb, Breshears, & Ford, 2013; Schuler & Smith, 1988). Because all these components exhibit their own dynamics and responses to drought, a comprehensive approach that integrates the different traits impacting overall tree gas exchange and water supply is key to understanding a tree's response to drought and predicting overall ecosystem resilience.

Previous investigations have focused on short‐term physiological responses influencing the hydraulic response of trees to drought (e.g., stomatal regulation and sapflow variation in the case of piñons; Pangle et al., 2015). Other studies have investigated the longer‐term adjustment in branch structure regulating the overall evaporative demand vs. sap supply (Feichtinger, Eilmann, Buchmann, & Rigling, 2015). Indeed, phenologically driven annual growth of xylem and needles, as well as leaf abscission, are influenced by exogenous (e.g., climate) and endogenous (e.g., carbon and nutrient status) factors (Manzoni, Vico, Thompson, Beyer, & Weih, 2015). Each year, additional evaporative structure is generated, potentially altering the plant hydraulic design and modulating the balance between demand (at the canopy level) and water supply (i.e., transported through the xylem). Under a steady‐state assumption, sap flow balances transpiration and the water potential gradient in xylem conduits can be expressed as (Tyree & Ewers, 1991):

(1) $$\frac{\text{\$\$d\$\$}\Psi_{X}}{\text{\$\$d\$\$}x}=\frac{E}{k_{L}}$$

where Ψ_*X*_ is the xylem water potential, *x* the length of the hydraulic pathway, *E* the evaporative flux density, and *k*_L_ the leaf‐specific conductivity, decomposed as *k*_L_ = *k*_S_·*A*_S_:*A*_L_ with *A*_L_ the total leaf area, *A*_S_ the total conducting sapwood area, and *k*_S_ the specific hydraulic conductivity. Structural changes that increase *k*_L_ mitigate the drop in $\frac{\text{\$\$d\$\$}\Psi_{X}}{\text{\$\$d\$\$}x}$ for the same level of transpiration *E*, which reduces the risk of cavitation (Cruiziat, Cochard, & Améglio, 2002). An increase in *k*_L_ can be achieved at constant hydraulic pathway (*k*_S_·*A*_S_) through a decrease in *A*_L_ or, at constant conductivity (*k*_S_), through a higher *A*_S_:*A*_L_ (Tyree & Ewers, 1991). A decrease in the maximal anatomical stomatal conductance (*g*_smax_) (Martínez‐Vilalta, Poyatos, Aguade, Retana, & Mencuccini, 2014) reduces *E* and thus can limit the drop of $\frac{\text{\$\$d\$\$}\Psi_{X}}{\text{\$\$d\$\$}x}$. Finally, variations in the annual elongation of branches might carry another hydraulic adjustment that can change the linear hydraulic resistivity (Poyatos et al., 2007). Analyzing the impact of drought and interannual climate variability on evaporative structure may thus be a key to understanding plant resilience to droughts.

We define “evaporative structure” as the needle traits constraining gas exchange between the tree and the atmosphere and thus participating in the regulation of carbon and water exchanges with the atmosphere. To assess the evaporative structure, numerous studies have considered a structural component approach by quantifying the functional ratio of total leaf area, *A*_L_, and normalized by total sapwood, *A*_S_ (presented as the Huber value = *A*_L_:*A*_S_ (Huber, 1928) or its converse *A*_S_:*A*_L_). Supporting a beneficial reduction in *k*_L_ to prevent cavitation (Equation (1)), increases in *A*_S_:*A*_L_ have been found along a geographical gradient of increasing dryness in both conifers (Callaway, DeLucia, & Schlesinger, 1994; DeLucia, Maherali, & Carey, 2000; Martínez‐Vilalta et al., 2009; Mencuccini & Bonosi, 2001; Whitehead, Edwards, & Jarvis, 1984) and angiosperms (Bucci et al., 2005; Carter & White, 2009; Gotsch et al., 2010; Li, Berninger, Koskela, & Sonninen, 2000). Only one instance of decreasing *A*_S_:*A*_L_ with increasing dryness has been reported in angiosperms from Eastern Europe (Sellin et al., 2013), while one study reported no variation along a Mediterranean gradient (Martin‐StPaul et al., 2013). As the fraction of active sap wood is not readily measurable, we here break down the multiple years of growth included in *A*_S_:*A*_L_ by focusing on the yearly addition of leaf area (LA) relative to yearly addition of sapwood area (SA), noted SA:LA. The response of this annual ratio to precipitation, which has only been studied in angiosperms at the most apical shoot, showed increasing SA:LA ratio with increasing dry conditions (Limousin et al., 2012; Martin‐StPaul et al., 2013). We expect the annual SA:LA in conifers to behave similarly to *A*_S_:*A*_L_ and increase with drought.

In branches, same evaporative structure and sapwood area but shorter branch length (*h*) will lead to a lower drop of water potential ∆Ψ_*X*_ at constant transpiration *E* (Equation (1), d*x* = *h*). A functional ratio that could help studying the influence of elongation on the evaporative structure response to drought is *A*_S_:*A*_L_/*h*, the inverse of the Huber Value corrected for the branch length. The annual adjustment of SA:LA/*d* (where *d* is the distance to tip) in response to drought might reduce the drop of water potential in new tissues. The annual shoot elongation of piñons branches is reduced under drought conditions (Adams et al., 2015; Grossiord et al., 2016); therefore, we expect SA:LA/*d* to increase with droughts.

Changes in the stomatal conductance also contribute to the variability of the evaporative structure of plants. Total stomatal conductance (*g*_s_) is the result of a dynamic response to various physiological and meteorological variables (e.g., light, VPD, abscisic acid, leaf water potential) and the anatomical component related to stomatal geometry and distribution, called maximum anatomical stomatal conductance*, g*_smax_ (Dow, Berry, & Bergmann, 2014). To date, studies have focused on change of *g*_smax_ in response to long‐term atmospheric CO_2_ concentration acclimation (Franks, Leitch, Ruszala, Hetherington, & Beerling, 2012; Franks et al., 2013, 2014), or on the theoretical framework for optimal use of the epidermal area for gas exchange (de Boer et al., 2016). Experimental drought has been shown to increase stomatal density and reduce stomatal size in angiosperms (Bosabalidis & Kofidis, 2002; Spence, Wu, Sharpe, & Clark, 1986; Xu & Zhou, 2008), which has been reported to correlate with an increase in *g*_smax_ (Franks, Drake, & Beerling, 2009).

We hypothesized that piñon trees, which display a relatively isohydric strategy (i.e., maintaining relatively constant leaf water potential, Ψ_L_, irrespective of soil water conditions; Limousin et al., 2013), would adjust their annual evaporative structure in response to soil moisture and VPD to reduce the drop of water potential in the xylem ($\frac{\text{\$\$d\$\$}\Psi_{X}}{\text{\$\$d\$\$}x}$). More precisely, we hypothesized that in years with low soil moisture and/or high VPD, piñons would (1) reduce needle length (*l*) and needle area; (2) increase the stomatal density and reduce the stomatal diameter, resulting in a decrease of maximum anatomical stomatal conductance *g*_smax_; and (3) increase the annual SA:LA and annual SA:LA/*d*.

To test these hypotheses, we analyzed the drought‐induced response of piñon pines over a 7‐year experiment in New Mexico that artificially modified soil moisture conditions, with three types of treatments: ambient, irrigated, and precipitation exclusion (so‐called droughted). Atmospheric VPD was similar across treatments, allowing us to decouple the effects of atmospheric dryness from soil water stress during multiple years, overcoming an issue for understanding long‐term ecosystems response over long time periods (Novick et al., 2016).

## 2 METHODOLOGY

### 2.1 Study site and experimental design

The study site is a mature piñon‐juniper woodland located at the Sevilleta Long‐Term Ecological Research Area LTER (34°23′11″N, 106°31′46″W; 1,911 m asl) in the Los Pinos Mountains of the Sevilleta National Wildlife Refuge. The climate record (25 years, 1991–2015) from the closest (2 km) meteorological station in the Long‐Term Ecological Research network (LTER, Cerro Montosa #42; http://sev.lternet.edu) indicates a mean annual precipitation of 355.3 mm with a standard deviation *SD* = 83.4 (Table 1) for the hydrological year taken from November to October. Precipitation in the region is bimodal with an average 18% of precipitation occurring during the winter (November to February) and 52% during the monsoon season (July to September). Mean annual (from January to December) maximum daily temperature is 18.8°C, ranging from 7.0°C in December to 29.8°C in July. Climate data at the experimental site were collected using a micrometeorological station centrally located in an open intercanopy area of the study site and stored on a CR‐10X datalogger. From 2007 to the end of 2013, precipitation was recorded with a Series 525 rain gauge (Texas Electronics, Dallas, TX), air temperature, and relative humidity with a Vaisala HMP45C sensor (Vaisala Oyj, Helsinki, Finland) (Table 1).

**Table 1:** Long‐term climatic statistics from LTER weather station (#42) and from field site met‐station

Period	Location	Dry season Max VPD (kPa)		Monsoon Max VPD (kPa)		Yearly PPT (mm)		Pre‐monsoon PPT (mm)		Monsoon PPT (mm)	
		Mean	*SD*	Mean	*SD*	Mean	*SD*	Mean	*SD*	Mean	*SD*
1991–2015	LTER #42	2.43	0.24	2.57	0.30	355.3	84.3	144.8	60.7	210.7	63.0
2007–2013	LTER #42	2.58	0.31	2.6	0.24	328.1	93.4	115.7	68.0	212.4	67.9
2007–2013	Field site	2.62	0.24	2.82	0.26	314.5	73.3	122.7	58.5	191.8	60.2
2007	Field site	2.35	–	2.92	–	323.7^a^	–	174.3^a^	–	149.3	–
2008	Field site	2.61	–	2.48	–	–	–	96.8	–	253.8	–
2009	Field site	2.35	–	2.87	–	281.7	–	105.9	–	175.8	–
2010	Field site	2.49	–	2.9	–	352.3	–	202.7	–	149.6	–
2011	Field site	2.87	–	3.07	–	163	–	34.5	–	128.5	–
2012	Field site	2.91	–	3.06	–	352.7	–	159.2	–	193.4	–
2013	Field site	2.8	–	2.42	–	377.4	–	85.3	–	292.1	–

*SD*, standard deviation.

Yearly PPT is the yearly cumulative precipitation from November 1st to October 31st, premonsoon PPT is from November 1st to June 30th, monsoon PPT from July 1st to October 31st.

a Value obtained from LTER (on 2008:2013, corr = .9651, *p* = .0018).

The experimental design consisted of three flat 40 × 40 m plots with different water treatments: (1) ambient conditions, (2) an artificial water addition from April to October, and (3) a precipitation exclusion (reduction of ~45 ± 1%). These 6‐year treatments spanned from the beginning of 2008 extending through the end of 2013, with the rain exclusion treatment starting in August 2007. Water addition dates were distributed from April to October with an average of ~19 mm per watering event resulting on annual addition rates of 57, 69.5, 112, 107, 95, and 95 mm/year. Further details of the water treatments and site infrastructures can be found in Pangle et al. (2012) and Plaut et al. (2012).

**Figure 1:**
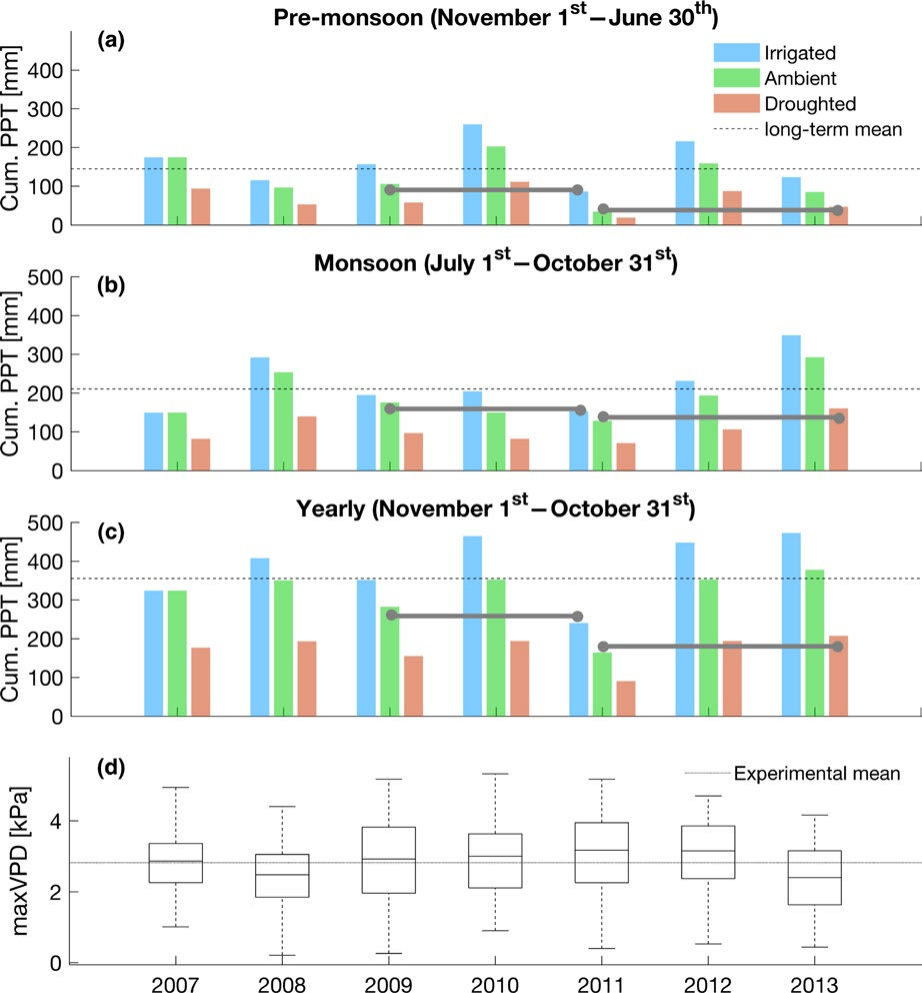
Climate during the years of the experiment and across treatments. (a) Cumulative precipitation during the pre‐monsoon period, (b) cumulative precipitation during the monsoon, (c) total yearly precipitation, initiating on November 1st, (d) distribution of maximum daily vapor pressure deficits during the monsoon. Dotted lines indicate the long‐term mean derived with LTER long‐term dataset. The continuous line indicates the mean during the experiment and measured at the experimental site. Horizontal gray segments visually identify two treatments that had similar seasonal water input but on different years

During the 6 years of the experiment, a wide range of climatic conditions occurred (Table 1, Figures S1 and S2). Four years (2009, 2010, 2011, 2012) had a cumulative monsoon precipitation below average: the irrigation treatment thus compensated for this low precipitation supply, the ambient treatment experienced natural drought conditions, while the droughted treatment faced extreme drought conditions (Figure 1). The year 2011 was extremely dry with premonsoon and monsoon precipitation approximately two (2σ) and one (1σ) standard deviations below the climatology, respectively. The mean maximum daily VPD at LTER site was below the long‐term VPD monsoon average, suggesting the absence of atmospheric drought at the field site with the exception of years 2011 and 2012, which had extremely high daily maximum VPD during the monsoon (approx. 1σ above the experimental mean) (Table 1, Figures S1 and S2).

### 2.2 Tree selection and sampling

Sample size for the irrigated, control, and droughted treatments were 10, 10, and 4 *P. edulis* Engelm trees, respectively (Tables S1 and S2). In May 2014, between 6 a.m. and 12 p.m., we collected three branches per tree so that at least the last 7 years of shoot elongation were included. All sampled branches were south‐facing and at the highest possible part of the tree crowns (between 2 and 4.5 m). They were carefully chosen to avoid possible local disturbances from previous twig collections used for leaf water potential measurements (Limousin et al., 2013). All branches were placed in plastic bags with a moist sponge and stored in ice‐coolers until their transportation to the laboratory in the afternoon for longer‐term storage in a freezer (−20°C).

### 2.3 Needle structure

**Figure 2:**
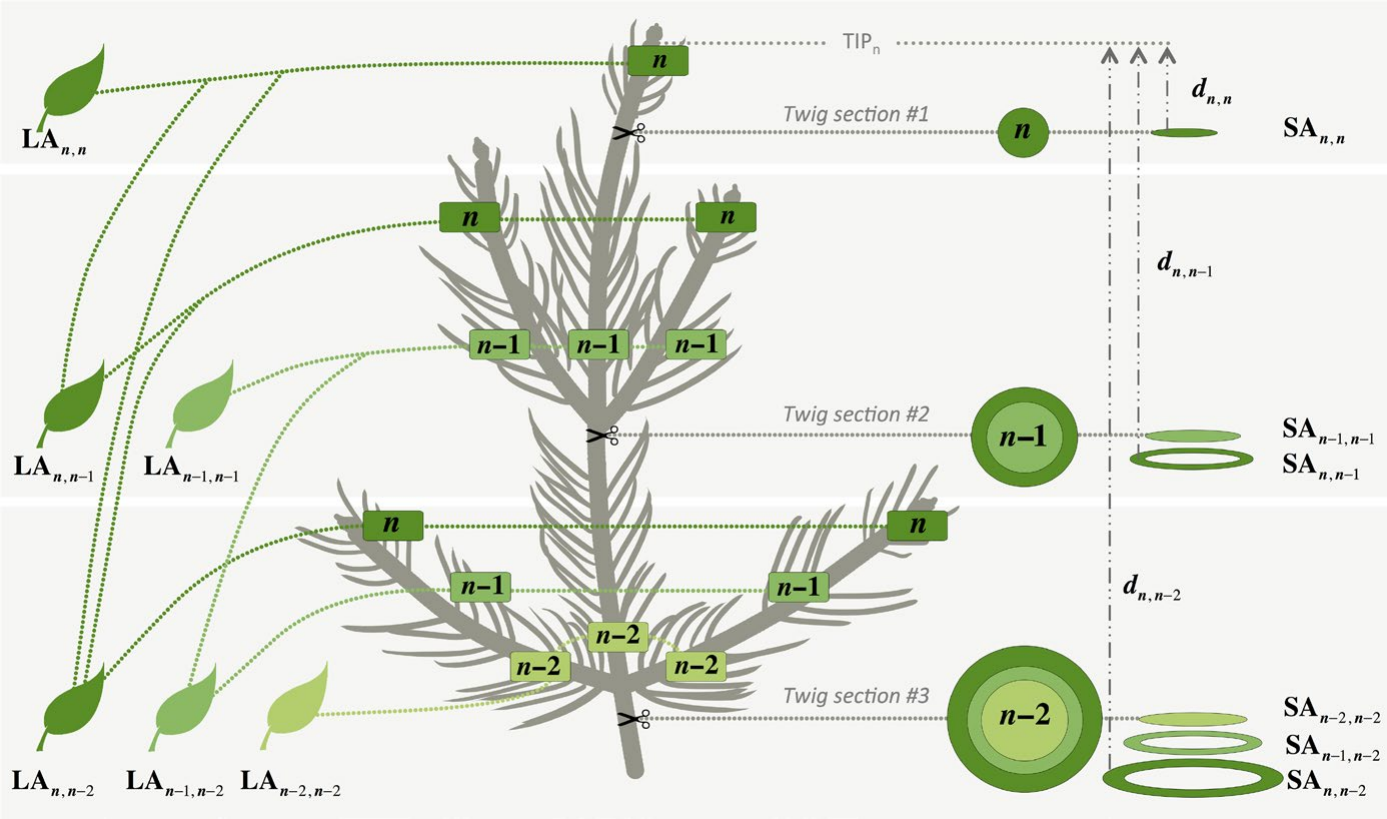
Scheme of the structural dissection. Different shades of green correspond to different years. *n* is the last year of growth present on the branch (here 2013). LA_*y*,Γ_ is the total leaf area of all needles formed in year *y* and that are between the branch tip and the twig section whose genesis occurred in year Γ. Similarly, SA_*y*,Γ_ is the xylem area that was added during year *y* and measured on a twig section whose genesis occurred in year Γ. *d*_*y*,Γ_ is the distance between the tip as of year *y* (TIP_*y*_) and the section where SA_*y*,Γ_ was measured (only 1 year shown for readability). For example: LA_*n*,*n*−1_ is the total leaf area of all needles formed in year *n* that are between the tip and twig section #2 (genesis in year *n *− 1); SA_*n*,*n*−1_ is the ring area formed in year n in the twig section #2 (genesis year *n *− 1); *d*_*n*,*n*−1_ is the length between SA_*n*,*n*−1_ and TIP_*n*_

Needles from the primary axis of each branch were removed and sorted by year of formation (Figure 2). The average number of years of needles on each branch was 5.5 with a standard deviation *SD *= 0.96 (ranging from 4 to 7 years). An average of 13.2 needles with *SD* = 3.1 (ranging from 1 to 15) were randomly selected for each year and pooled to create a yearly subsample. On each needle of these subsamples, we counted the number of stomatal rows in the adaxial (*R*_ad_, with a precision of ±0.5) and abaxial faces (*R*_ab_, ±0.5) under a microscope (Nikon SMZ‐U; Nikon, Tokyo, Japan) at a magnification of ×5. Then, we scanned the adaxial face of these needles on a flat‐bed scanner at a resolution of 1,200 dpi. We measured needle length (*l*) and width in mm (*w*) with a precision of ±0.02 mm in Adobe Illustrator CS5 15.0.0 (Adobe Systems Inc., San Jose, CA, USA) for a total of 4,918 needles. The needle adaxial area (*A*_ad_) was measured with a precision of ±0.01 mm^2^ with Adobe Photoshop CS5 12.0 (Adobe Systems Inc.) for 20% of the needles. Using these measurements, we used linear regression to estimated *A*_ad_ for all needles (*A*_ad_ **= γ + α·*l*·*w*;* R* ^2^ = .966; Figure S3). From the subsamples of needles used for measuring *w* and *l*, we randomly selected 1–5 needles (an average of 4.8 needles, *SD* = 0.6) to measure stomatal features. From each needle, adaxial face imprints at the widest part of the needle (middle section) were produced using nail polish and tape (Voleníková & Tichá, 2001). From these imprints, we measured the mean linear density of stomata in rows in each subsample ($\bar{D_{\mathrm{lin}}}$, stomata/mm) and maximal stomatal diameter (*d*_S_, μm) with a precision of 4 μm under a microscope (Olympus, BX50; Olympus Austria Corp., Vienna, Austria) at a magnification of ×400, with a field of view of 8.5 mm^2^ (Camargo & Marenco, 2011). Stomatal density of each needle (*D*_s_, stomata number/mm^2^) was then calculated considering *A*_ad_ ≈ *A*_ab_ (i.e., neglecting the slight curvature of the abaxial face):

(2) $$D_{s}=\frac{\bar{D_{\mathrm{lin}}}l\left(R_{\mathrm{ad}}+R_{\mathrm{ab}}\right)}{2A_{\mathrm{ad}}}$$

Maximal anatomical stomatal conductance, *g*_smax_ (mol m^−2^ s^−1^), has been described as a long‐term adaptation parameter (de Boer et al., 2011). It can be related to stomatal conductance and is a function of *d*_S_ and *D*_s_ (Lammertsma et al., 2011) which are two potential parameters for the study of drought acclimation (Hepworth, Doheny‐Adams, Hunt, Cameron, & Gray, 2015).

A yearly maximal anatomical stomatal conductance was derived for each branch using the formula (Dow, Bergmann, & Berry, 2014):

(3) $$g_{\mathrm{smax}}\left(y\right)=\frac{d{\bar{a_{\max}}}^{y}}{v\left({\overset{¯}{p}}^{y}+\frac{\pi }{2}\sqrt{{\bar{a_{\max}}}^{y}/\pi }\right)}{\bar{D_{s}}}^{y}$$

where ${\bar{D_{s}}}^{y}$ is the mean *D*_s_ for year *y* (stomata number/mm^2^), ${\bar{a_{\max}}}^{y}$ is the mean maximum stomatal pore area for year *y* (μm^2^), *d* is the diffusivity of water vapor in air (m^2^/s), *v* is the molar volume of air (m^3^/mol), and *p* is the pore depth (μm). *d* and *v* were computed at 25°C. In line with Mitton, Grant, and Yoshino (1998), who found similar dimensions for the length and width of *P. edulis* (difference of ~15%), we observed stomata with circular shape. We therefore approximated stomata as a half‐sphere, so that $\overset{¯}{p}=\bar{d_{S}}/2$ and $\bar{a_{\max}}=\pi \cdot {\left(\bar{d_{S}}/2\right)}^{2}$.

### 2.4 Sapwood

For measurement of ring area variables, we cut at least three segments of twig (~1–3 cm long) on one branch per tree (three branches per tree for the droughted treatment), along the primary axis for measurement of wood anatomical variables (Figure 2). From the most distal extremity of each segment, thin sections (20 μm thickness) were produced using a sliding microtome (WSL, Lab‐microtome, Switzerland). Using a digital camera (Canon EOS‐650D), images were taken from each thin section under a microscope (Olympus BX41) with a resolution of 2.36 pixels/μm. In each image, individual growth rings were identified and their ring area (SA) determined using ROXAS 2.0 (von Arx & Carrer, 2014) with Image‐Pro Plus 6.1 (Media Cybernetics, Silver Spring, MA, USA). Due to the small number of years, correct ring dating could only be made visually, but not statistically cross‐validated. This procedure suggested the existence of missing rings only for the extremely dry year 2011 (Figure S4).

### 2.5 Functional ratios

We used annual SA:LA to understand the trend in interannual variation and its drought response. Because annual SA and annual LA can be measured from different elongation segments of the branch (Figure 2), we clarify by noting SA_*y*,Γ_ the xylem area (in cm^2^) that was added during year *y* and measured on a twig section whose genesis occurred in year Γ (the innermost ring of this wood section). In other words, *y* gives the temporal information of when the SA was formed and Γ gives the spatial information of where SA was measured on the branch. Similarly, we note LA_*y*,Γ_ the total needle surface area (m^2^) formed in year *y* that is between the branch's tip and the twig section whose genesis occurred in year Γ. As a ring of year *y* is measured on a section whose oldest ring is Γ, Γ ≤ *y* holds (Figure 2). We then calculated the annual ratios SA:LA_*y*,Γ_ (cm^2^/m^2^):

(4) $${\text{\$\$SA:LA\$\$}}_{y,\Gamma }=\frac{{\text{\$\$SA\$\$}}_{y,\Gamma }}{{\text{\$\$LA\$\$}}_{y,\Gamma }}$$

After discarding the apical values corresponding to the innermost ring of each twig section (primary xylem), we averaged for each branch across all Γ, that is, across all wood sections analyzed on each branch (Appendix S1), to obtain one value per year per branch:

(5) $$\bar{{\text{\$\$SA:LA\$\$}}_{y}}={\bar{{\text{\$\$SA:LA\$\$}}_{y,\Gamma }}}^{\Gamma }$$

We further emphasize that this is different from total sapwood area (*A*_S_) divided by total leaf area (*A*_L_) because SA:LA represents the relative annual change. Indeed, it is not possible to retrospectively measure changes in total active sap area throughout the years, nor it is to retrospectively estimate *A*_L_, and thus we only focus on the annual changes and their trends.

We finally derived the yearly ratio of SA:LA/*d* (m^2^/m^3^), which integrates size‐related structural change of the branch within the framework of a hydraulic model (Tyree & Ewers, 1991). Similarly, we discard the apical values and obtain one value per year and per branch:

(6) $$\bar{\text{\$\$SA:LA\$\$}/d_{y}}={\bar{{\text{\$\$SA:LA\$\$}}_{y,\Gamma }/d_{y,\Gamma }}}^{\Gamma }$$

where *d*_*y*,Γ_ is the distance between the tip as of year *y* and the wood section where ${\text{\$\$SA:LA\$\$}}_{y,\Gamma }$ was measured (Figure 2, Appendix S2).

### 2.6 Statistical analysis

For each year, variables were compared between the three treatments using a two‐sided Wilcoxon test (i.e., three comparisons for 7 years of data). Within treatments, we tested whether needle structure for any specific year differed from the long‐term mean of all 7 years, using a two‐sample nonparametric Kolmogorov–Smirnov test. Regardless of the position on the branch (Γ), we also compared across treatments the linear relationship between ${\text{\$\$SA\$\$}}_{y,\Gamma }$ and ${\text{\$\$LA\$\$}}_{y,\Gamma }$ for the “extreme year” (year 2011 only) and “average years” (all experimental years except 2011). Relationships were obtained using linear regressions (with intercept = 0), and comparison of slopes was made using a bootstrap (Efron & Tibshirani, 1993). For all statistical tests, we used a significance level of α = 0.05 (unless otherwise noted). These analyses were completed in Matlab (R2016a; The MathWorks, Inc., Natick, MA).

We investigated the potential effects of (1) number of days with maximum VPD >4.5 kPa during the dry season (MAMJ) and the monsoon season (JASO) and (2) cumulative precipitation during premonsoon (November to June) and monsoon (July to October, JASO), with needle length, $\bar{\text{\$\$SA:LA\$\$}}$ and $\bar{\text{\$\$SA:LA\$\$}/d}$ using linear mixed effect models (LMEM) (Zuur, Ieno, Walker, Saveliev, & Smith, 2009). The choice of the VPD threshold was established after trying different values, and 4.5 kPa was the value that explained the highest variability. Precipitation and VPD periods were used in the LMEM as fixed effects and random effects to allow for different responses across treatments, using treatment as the grouping variable. To identify the best combination of predictors in our LMEM, we used a structured search approach (Tables S3 and S4) using the Akaike Information Criterion (AIC) and ANOVA tests, jointly with knowledge about physiological responses (e.g., precipitation is expected to positively affect needle length). In order to further guard against violating the underlying LMEM assumption of normality of residuals and coefficients, we confirmed the results using a bootstrapped nonparametric approach (Efron & Tibshirani, 1993). Similarly, rather than χ^2^‐tests, we used bootstrapping for comparing coefficients between treatments (5% level). Finally, simple linear regressions were run on the best predictors. These analyses were conducted using R (3.3.1, R Core Team, 2016) with the packages “stats” and “nlme” (Pinheiro et al., 2008).

## 3 RESULTS

### 3.1 Needle structure

**Figure 3:**
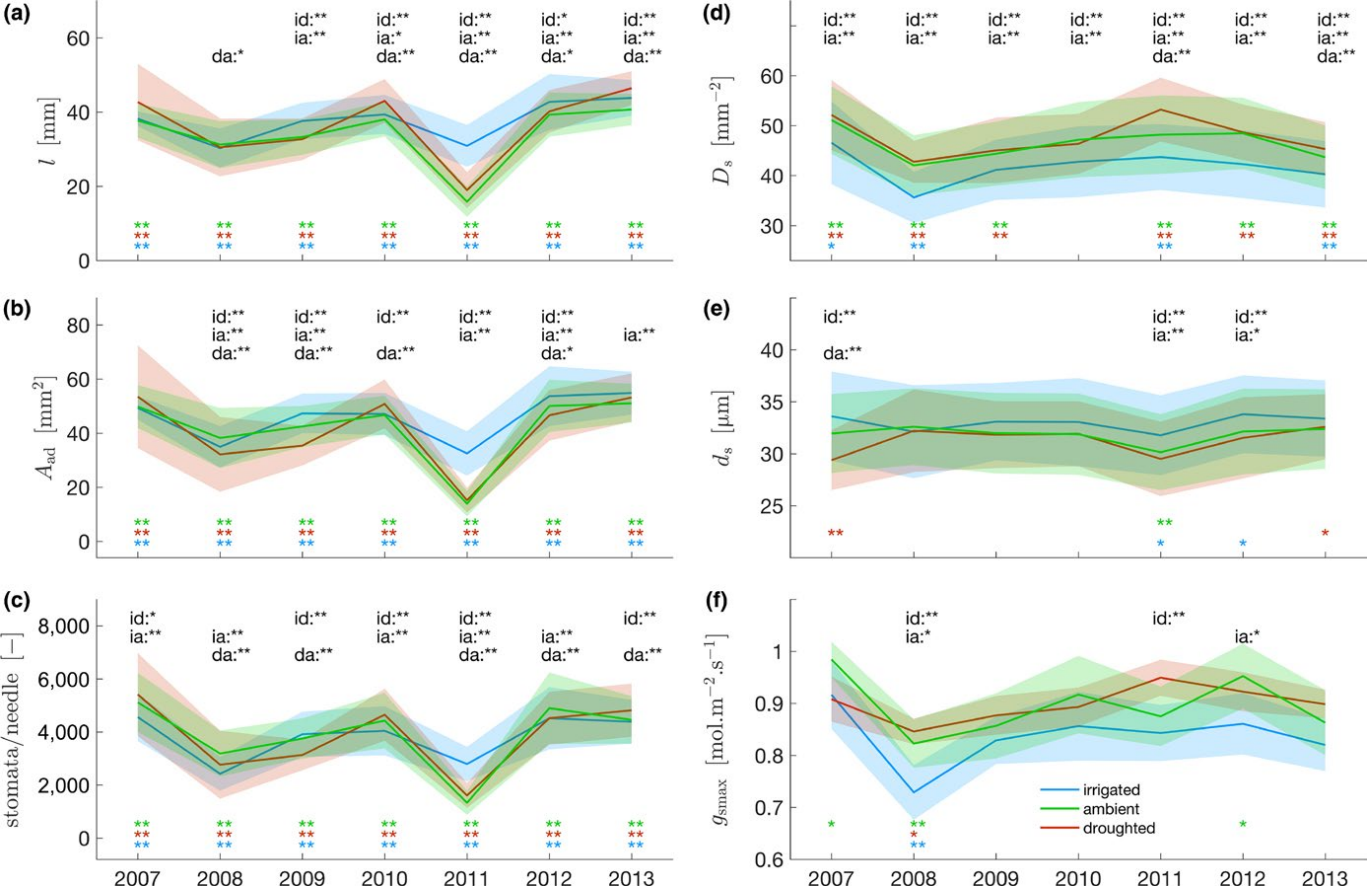
Time series of needle structure parameters. (a) Needle's length, (b) needle's area, (c) number of stomata per needle, (d) stomatal density, (e) stomatal diameter, (f) maximal anatomical stomatal conductance (*g*_smax_). Solid lines are the means per treatment, and shading are the standard deviations. Wilcoxon tests were performed to determine statistical difference in the yearly median across treatment, reported in the upper part under id, irrigated/droughted; ia, irrigated/ambient; da, droughted/ambient. For each individual treatment, Kolmogorov–Smirnov tests were performed to determine statistical difference in the distribution of yearly population against population obtain from pooling all years of the treatment, reported in the lower part and following the color code. Difference at the 5% level: *, at the 1% level: **

Needle structural parameters exhibited different responses to drought across treatments and years. In 2009, one year into the experiment, the branches of irrigated trees developed longer needles with larger areas than ambient trees and droughted trees (Figure 3a,b). In each treatment, median annual needle length and area, and number of stomata per needle showed significant variation compared to the median interannual value over the entire duration of the experiment. This variability was lowest in irrigated trees. During the driest year of the experiment (2011), needle length (*l*), needle area (*A*_ad_ + *A*_ab_), and number of stomata per needle decreased by 50% in ambient and droughted trees compared to the previous year, while irrigated trees showed a smaller decrease (10%–20%) (Figure 3a,c). In 2012, with rainfall closer to the mean, these values were again similar to those in 2010. Overall, *l* was positively associated with *A*_ad_ + *A*_ab_ and the number of stomata per needle (*r *=* *.96, *r *=* *.86, respectively, with *p *<* *.001). Stomatal density (*D*_S_) remained significantly smaller for the irrigated treatment throughout the experiment (Figure 3d). Compared to *l*,* A*_ad_ + *A*_ab_, and stomata per needle, stomatal density *D*_S_ exhibited less interannual variation within treatments, although 2008 and 2013 were significantly lower and 2011 significantly higher than the long‐term mean for all treatments (Figure 3d). Stomatal diameter (*d*_S_) and maximum anatomical stomatal conductance (g_smax_) showed little variation across treatments and little variability between years for the duration of the experiment. Irrigated trees showed higher *d*_S_, but these differences were only significant in 2011 and 2012 (Figure 3e). g_smax_ showed little significant differences across treatments or significant variability between years (*p *>* *.05, Figure 3f). On average, g_smax_ remained lower for trees of the irrigated treatment, while trees from the droughted and ambient treatments were similar (Figure 3f). This lower g_smax_ was primarily explained by the reduced density of stomata (*D*_S_) in the irrigated treatment.

**Figure 4:**
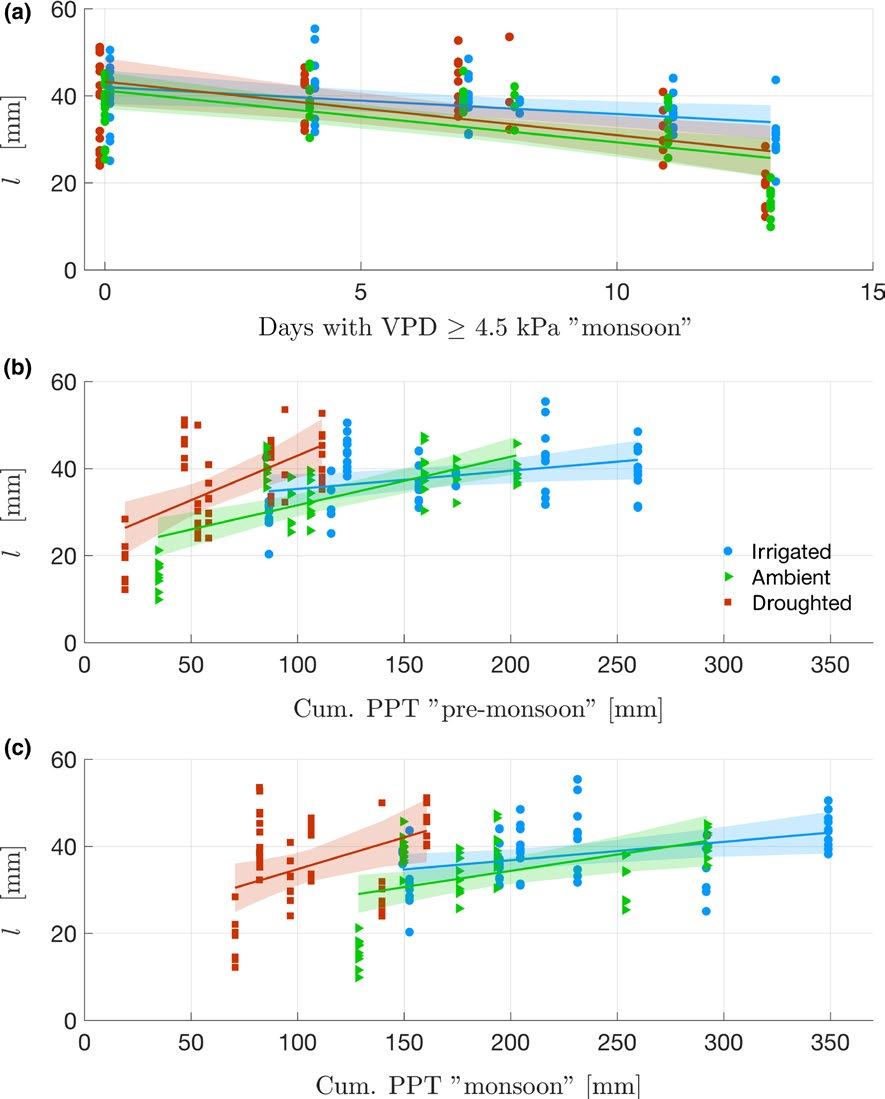
Relation between number of days with maximum VPD > 4.5 kPa and cumulative precipitation (Cum. PPT) with needle length on the period 2007–2013. (a) Needle length (*l*) versus number of days of VPD > 4.5 kPa during the dry season. (b) *l* versus cumulative precipitation during premonsoon (c) *l* versus cumulative precipitation during monsoon. Each dot represents one year average from one branch (N=173). Colored lines are regressions for each treatment. Shaded areas are confidence intervals of each regression. Results of linear regressions are found S6a For readability, an artificial abscissa offset was added for droughted and ambient treatments in (a)

For each treatment, needle length increased with both premonsoon and monsoonal precipitation but decreased with VPD (Figure 4, Table S6). Using linear mixed effect models (LMEM), the variance of needle length was explained using different combinations of climate predictors (two precipitation periods and two VPD periods, total of four combinations). Including premonsoon precipitation as predictor always increased the model fit with respect to the same model without this variable. Because VPD and monsoon precipitation are strongly correlated (*r *=* *−.89, *p *=* *.008, Table S5), no improvement was achieved when using both of these predictors compared to only one. Among all the models tested, the needle length, *l,* was best fitted (based on AIC, Table 2a) by premonsoon precipitation (*p *<* *.05) and monsoon precipitation (*p *<* *.05) as fixed effects (explaining 32% of the variance; Johnson, 2014). As expected, for each treatment, needle length decreased with increasing VPD and it increased with both precipitation periods for all models (Table 3a). A nonparametric bootstrap test confirmed that the interannual response of *l* to precipitation change was higher on the irrigated treatment than the ambient treatment, itself smaller than the droughted treatment (βppt‐irrigated < βppt‐ambient < βppt‐droughted, methods in Appendix S3 and R code in Appendix S4 and results in Table S7a). Needle length did not exhibit any significant response to VPD across treatments (Table S7a), except during the very dry year of 2011 (Figure 3a), emphasizing that VPD affected all treatments similarly, independently of soil moisture.

**Table 2:** Results of mixed linear effect models of the form *Y* = β0 + β1·*X*1 + β2·*X*2 with *Y the modeled variable*,* X*1 and *X*2 standardized climate predictors (yearly value minus experimental mean divided by standard deviation)

*Y*	Climate predictors		Fixed effect			Random effect	AIC	$r_{\left(m\right)}^{2}$	$r_{\left(c\right)}^{2}$
			Value	*SE*	*p*‐Value	*SD*			
**a. ** ***l***	Intercept	β0	31.7	2.38	<.001^a^	3.21	1,172	.43	.77
	PPT: pre‐monsoon std	β1	0.1	0.04	.009^a^	0.06			
	VPD: monsoon std	β2	−0.86	0.18	<.001^a^	0.26			
	Intercept	β0	6.8	6.1	.27	9.97	1,151	.32	.94
	PPT: pre‐monsoon std	β1	0.14	0.06	.014^a^	0.09			
	PPT: monsoon std	β2	0.11	0.04	.005^a^	0.06			
**b.** $\bar{\text{SA:LA}}$	Intercept	β0	1.42	0.06	<.001^a^	3.8 e‐2	355	.32	.33
	PPT: pre‐monsoon std	β1	0.31	0.07	<.001^a^	7.2 e‐2			
	VPD: monsoon std	β2	−0.3	0.05	<.001^a^	3.8 e‐5			
	Intercept	β0	1.53	0.25	<.001^a^	0.41	367	.37	.59
	PPT: pre‐monsoon std	β1	0.37	0.1	<.001^a^	0.12			
	PPT: monsoon std	β2	0.41	0.15	<.01^a^	0.22			
**c.** $\bar{\text{SA:LA}/d}$	Intercept	β0	1.7 e‐3	0.1 e‐3	<.001^a^	9.5 e‐7	−1,644	.16	.16
	PPT: pre‐monsoon std	β1	0.3 e‐3	0.1 e‐3	<.001^a^	12.8 e‐7			
	VPD: monsoon std	β2	−0.4 e‐3	0.1 e‐3	<.001^a^	8.3 e‐7			
	Intercept	β0	1.69 e‐3	0.1 e‐3	<.001^a^	0.5 e‐5	−1,635	.11	.12
	PPT: pre‐monsoon std	β1	0.31 e‐3	0.1 e‐3	.021	12.2 e‐5			
	PPT: monsoon std	β2	0.36 e‐3	0.1 e‐3	.018	3.8 e‐5			

Intercept, *X*1, and *X*2 are fixed and random effects, with β0, β1, and β2 their respective coefficients. Needle length (*l*) in mm. $\bar{\text{\$\$SA:LA\$\$}}$ in cm^2^/m^2^. $\bar{\text{\$\$SA:LA\$\$}/d}$ in m^2^/m^3^. $r_{\left(m\right)}^{2}$ and $r_{\left(c\right)}^{2}$ refer, respectively, to marginal *R* ^2^ and conditional *R* ^2^ (Johnson, 2014).

a Notifies a *p* < .05.

**Table 3:** Equations from mixed linear effect model for *Y = *β0 + β1·*X*1 + β2·*X*2 with *X*1 and *X*2 climate variables taken as fixed and random effects

Treatment	β0	βl‐treatment	β2‐treatment	*n*
**a. ** ***l***				
		PPT: pre‐monsoon^a^	VPD: monsoon	
Irrigated	34.7	0.042	−0.58	58
Ambient	28.8	0.093	−0.96	54
Droughted	31.5	0.161	−1.04	61
		PPT: pre‐monsoon^a^	PPT: monsoon^a^	
Irrigated	18	0.051	0.05	58
Ambient	0.4	0.126	0.096	54
Droughted	2	0.236	0.171	61
**b.** $\bar{\text{SA:LA}}$				
		PPT: pre‐monsoon	VPD: monsoon	
Irrigated	1.35	4.0 e‐3	−0.062	48
Ambient	1.23	5.0 e‐3	−0.062	58
Droughted	1.19	5.0 e‐3	−0.062	57
		PPT: pre‐monsoon	PPT: monsoon	
Irrigated	0.11	4.2 e‐3	3.8 e‐3	48
Ambient	−0.09	6.5 e‐3	4.1 e‐3	58
Droughted	−0.21	6.5 e‐3	8.0 e‐3	57
**c.** $\bar{\text{SA:LA}/d}$				
		PPT: pre‐monsoon	VPD: monsoon	
Irrigated	15.7 e‐4	5.3 e‐6	−80.1 e‐6	48
Ambient	15.7 e‐4	5.3 e‐6	−80.1 e‐6	58
Droughted	15.7 e‐4	5.3 e‐6	−80.1 e‐6	57
		PPT: pre‐monsoon	PPT: monsoon	
Irrigated	5.7 e‐4	4.2 e‐6	3.6 e‐6	48
Ambient	3.8 e‐4	5.8 e‐6	3.6 e‐6	58
Droughted	5.7 e‐4	4.4 e‐6	3.6 e‐6	57

Needle length (*l*) in mm. $\bar{\text{\$\$SA:LA\$\$}}$ in cm^2^/m^2^. $\bar{\text{\$\$SA:LA\$\$}/d}$ in m^2^/m^3^. *n* the sample size of each treatment.

a The three treatment slopes are significantly different at the 5% level.

### 3.2 Functional ratios

**Figure 5:**
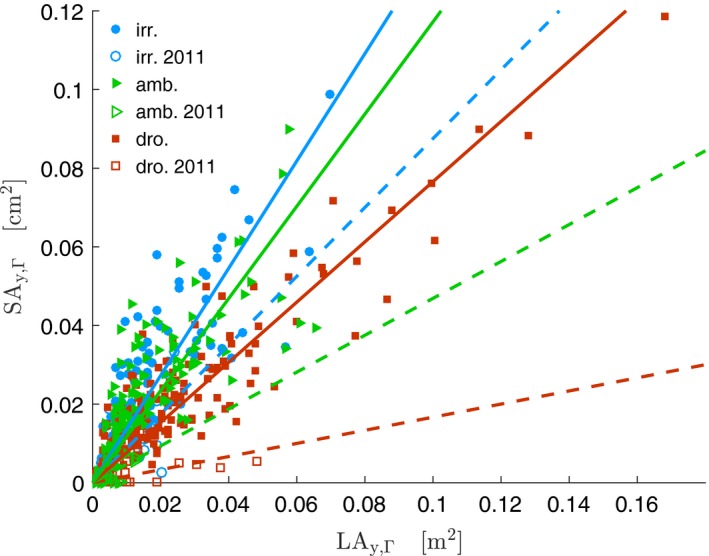
Relation between SA_*y*,Γ_ and LA_*y*,Γ_ on the period 2008‐2013 (N=440, includinc apical values). Filled symbols represent “average years” of the experiment and solid lines are the regressions for each treatment (S8a). Empty symbols represent the “extreme year” (2011), and dashed lines are the regressions for each treatment (S8b). For a zoomed version, see S5

For “average years,” the linear regressions of SA_*y*,Γ_ on LA_*y*,Γ_, led to significantly larger slopes (SA:LA_*y*,Γ_) on the irrigated and ambient treatments compared to the droughted treatment (Figure 5, Table S8a). For the “extreme year” (2011), all slopes were significantly different and decreased with dryness (Figure 5, S8b).

**Figure 6:**
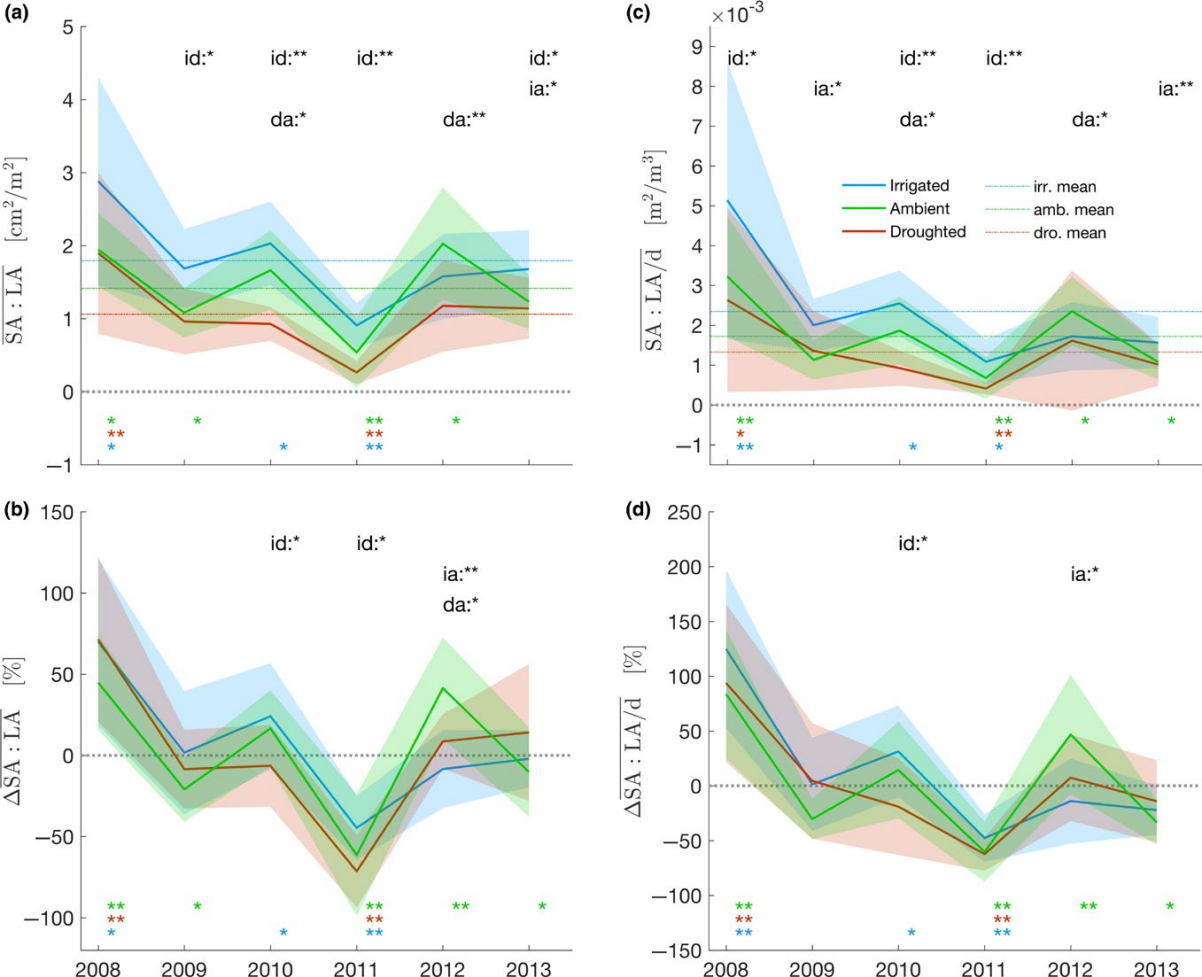
Time series of the functional ratios and their variations around the mean (2008–2013). (a,c) $\bar{\text{\$\$SA:LA\$\$}}$ and $\bar{\text{\$\$SA:LA\$\$}/d}$ pooled by treatments, dashed lines indicating the mean during the experiment. (c,d) percentage of variation of $\bar{\text{\$\$SA:LA\$\$}}$ and $\bar{\text{\$\$SA:LA\$\$}/d}$ around the mean of each treatment, pooled by treatments. For (a) and (c), dotted lines are the mean per treatment. Shading are standard deviations (±σ). Similarly to Figure 3, Wilcoxon tests and Kolmogorov–Smirnov tests were reported

The yearly branch average $\bar{\text{\$\$SA:LA\$\$}}$ was mostly higher in the irrigated treatment compared to the ambient treatment, which was higher than the droughted treatment (Figure 6a). Mean $\bar{\text{\$\$SA:LA\$\$}}$ values for the duration of the experiment (${\bar{\text{\$\$SA:LA\$\$}}}_{2008:2013}$) were 1.79, 1.41, 1.06 cm^2^/m^2^ (*SD*: 0.64, 0.57, 0.52) for the irrigated, ambient, and droughted treatments, respectively (Figure 6a). When taking the percentage of variation of ${\bar{\text{\$\$SA:LA\$\$}}}_{y}$ around its treatment mean, the difference between treatments collapsed and resulted in very few significant differences for ${\bar{\text{\$\$SA:LA\$\$}}}_{y}$ (Figure 6b), thus indicating a linear response. The largest positive variations of $\bar{\text{\$\$SA:LA\$\$}}$ occurred for year 2008 (+71% in the droughted treatment) and the largest negative variation in the extremely dry year of 2011 (−71% in the droughted treatment) (Figure 6b).

Similarly, $\bar{\text{\$\$SA:LA\$\$}/d}$ was higher in the irrigated treatment compared to the ambient and droughted treatments (Figure 6c). Mean ${\bar{\text{\$\$SA:LA\$\$}/d}}_{2008:2013}$ were 2.34 × 10^−3^, 1.72 × 10^−3^, 1.33 × 10^−3^ m^2^/m^3^ (*SD*: 1.45 × 10^−3^, 0.95 × 10^−3^, 0.76 × 10^−3^) for the irrigated, ambient, and droughted treatments, respectively (Figure 6c). The percentage of variation of ${\bar{\text{\$\$SA:LA\$\$}/d}}_{y}$ around its mean resulted in almost no difference for ${\bar{\text{\$\$SA:LA\$\$}/d}}_{y}$ between treatments (Figure 6d). The largest positive variations occurred for year 2008 (+125% in the irrigated treatment) and the largest negative variation in the extremely dry year of 2011 (−62% in the droughted treatment) (Figure 6d).

**Figure 7:**
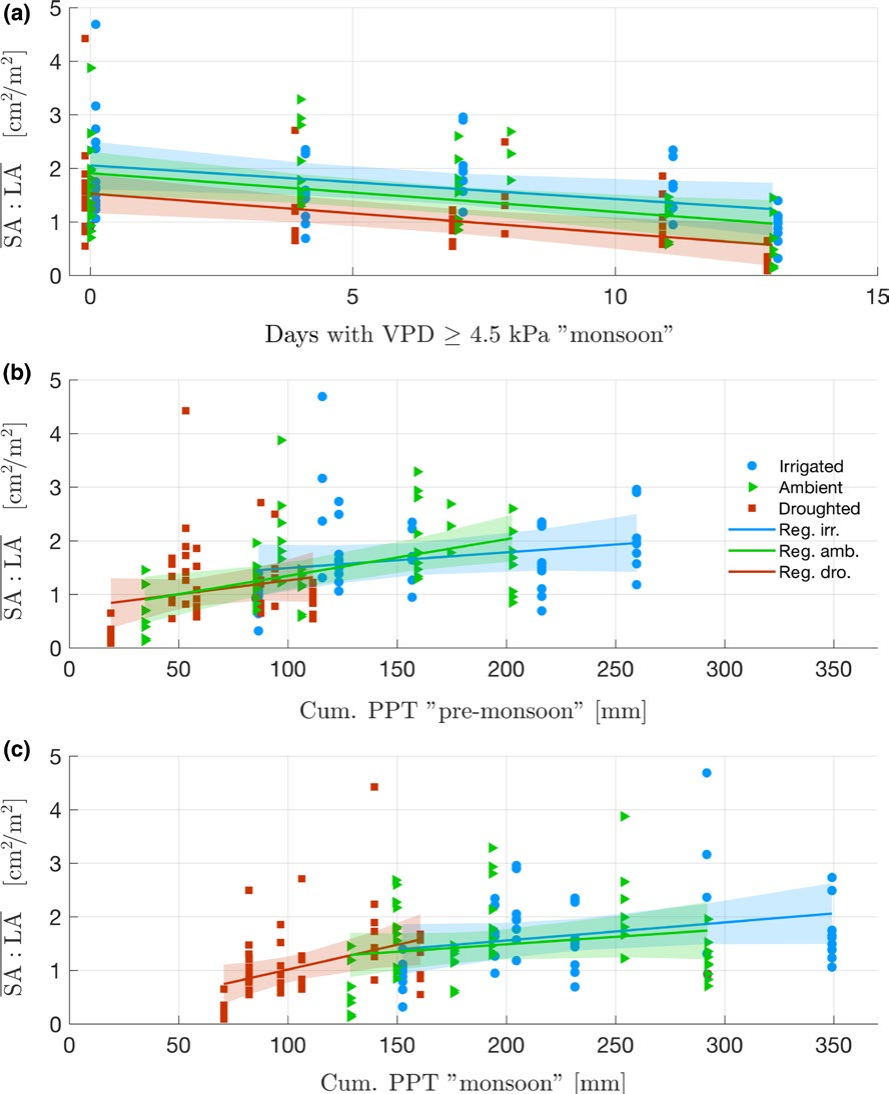
Relation between climate variables and $\bar{\text{\$\$SA:LA\$\$}}$ calculated for each branch on the period 2007‐2013 (N=163). (a) $\bar{\text{\$\$SA:LA\$\$}}$ versus number of days of VPD > 4.5 kPa during monsoon. (b) $\bar{\text{\$\$SA:LA\$\$}}$ versus cumulative precipitation during premonsoon. (c) $\bar{\text{\$\$SA:LA\$\$}}$ versus cumulative precipitation during monsoon. Colored lines are regressions for each treatment. Shaded areas are confidence intervals of each regression. Results of linear regressions are found in S6b For readability, an artificial abscissa offset was added for droughted and ambient treatments in (a)

**Figure 8:**
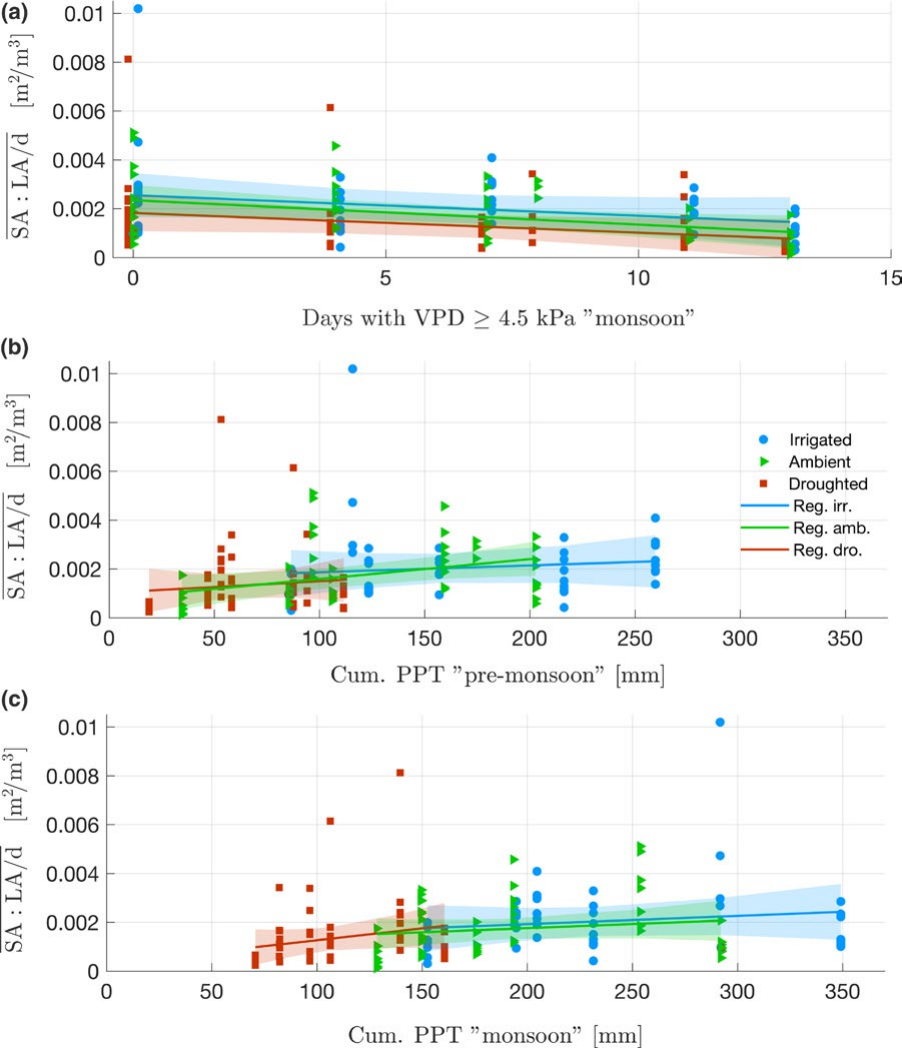
Relation between climate variables and $\bar{\text{\$\$SA:LA\$\$}/d}$ calculated for each branch on the period 2007‐2013 (N=163). (a) $\bar{\text{\$\$SA:LA\$\$}/d}$ versus number of days of VPD > 4.5kPa during monsoon. (b) $\bar{\text{\$\$SA:LA\$\$}/d}$ versus cumulative precipitation during premonsoon. (c) $\bar{\text{\$\$SA:LA\$\$}/d}$ versus cumulative precipitation during monsoon. Colored lines are regressions for each treatment. Shaded areas are confidence intervals of each regression. Results of linear regressions are found in S6c. For readability, an artificial abscissa offset was added for droughted and ambient treatments in (a)

For each treatment, similarly to needle length, $\bar{\text{\$\$SA:LA\$\$}}$ and $\bar{\text{\$\$SA:LA\$\$}/d}$ increased with precipitation and decreased with VPD (respectively Figure 7, Table S6b and Figure 8, Table S6c). Using LMEM, the ratios were best fitted (based on AIC, Table 2b,c) by premonsoon precipitation (*p *<* *.05) and monsoon VPD (*p *<* *.05) as fixed effects (explaining, respectively, 32% and 16% of the variance, $r_{\left(m\right)}^{2}$; Johnson, 2014). The random effect did not improve the fitting ($r_{\left(c\right)}^{2}$ ≈ $r_{\left(m\right)}^{2}$; Johnson, 2014) (Table 2b,c) and modeling the logarithm of the ratios led to the same best fits (Table 3b,c). The bootstrap confirmed the LMEM results (Table S7b,c), except for the significance of premonsoon precipitation in predicting $\bar{\text{\$\$SA:LA\$\$}/d}$ in two treatments. Response of $\bar{\text{\$\$SA:LA\$\$}}$ and $\bar{\text{\$\$SA:LA\$\$}/d}$ to climate predictors did not change significantly across treatments (*p *>* *.05, Table S7b,c). Overall, the effect of VPD and precipitation periods seemed to affect both ratios with similar magnitude across treatments (comparing coefficients after standardizing climate predictors).

## 4 DISCUSSION

In our 7‐year experiment, we showed that yearly variation in evaporative structure of piñon pines was driven by changes in soil moisture across treatments. Specifically, needle length, area, and stomatal density of needles all decreased with drier soil, whereas neither stomatal diameter nor maximal anatomical stomatal conductance varied significantly. Needle length, SA:LA and SA:LA/*d* differed across treatments and correlated positively with precipitation, but only needle length responded differently across treatments—same water input resulting in significantly different length. Atmospheric drought, through high VPD, had an impact only when cooccurring with extreme soil drought, as observed in 2011. In branches, the response of the new evaporative structure to drought is immediate and does not support a reduction of the water potential gradient in newly built xylem.

### 4.1 Climate impact on evaporative structure

Because plant species that behave more isohydrically react to low moisture levels by reducing water use even at the expense of lower carbon gain through stomatal regulation (Limousin et al., 2013), we hypothesized that piñon pines would adjust their evaporative structure under low soil moisture by increasing stomatal density (*D*_S_) and reducing stomatal diameter (*d*_S_). Our results confirmed our hypothesis and piñons increased *D*_S_ and slightly decreased *d*_S_ in response to low soil moisture. Similar trends of higher *D*_S_ under drought were also found in young *Pinus taeda* L. in a dry region of South Central US (Bilan & Knauf, 1974) and in angiosperms (Clifford et al., 1995; Quarrie & Jones, 1977). However, the opposite trend has also been observed for some angiosperm species which responded to low soil moisture by reducing both *D*_S_ and *d*_S_ (Doheny‐Adams, Hunt, Franks, Beerling, & Gray, 2012; Franks et al., 2009; Taylor et al., 2012). The large variety of responses among species suggests that plasticity in stomatal morphology might serve different goals for different species (Franks & Farquhar, 2007). In the case of the relatively isohydric piñons experiencing long‐term water stress, it is beneficial to reduce stomatal size to maintain an efficient and rapid control of stomatal aperture and closure (Drake, Froend, & Franks, 2013; Franks et al., 2012). The reduction in pore size often correlates with increased stomatal density (Franks et al., 2009), at the expense of a higher energy cost associated with the maintenance and operations of individual stomata (Assmann & Zeiger, 1987). Contrary to our hypothesis, the variations in *D*_S_ and *d*_S_ mostly offset each other and thus did not result in a significant change in *g*_smax_ across treatments and years, so that this parameter was not sensitive to yearly changes in climate. This result contrasts with the response of the more anisohydric *Eucalyptus globulus* seedlings which have shown to increase *g*_smax_ with rainfall (Franks et al., 2009). As suggested by de Boer et al. (2016), gymnosperm might not benefit from increasing *g*_smax_ due to inherently low leaf water transport capacity. Relatively isohydric piñons might not benefit either from a decrease in *g*_smax_ as they have good control of stomatal closure.

In conifers, the dates of needle emergence and total needle length correlate positively with soil moisture during the growing season (Dobbertin et al., 2010; Raison, Myers, & Benson, 1992; Sheffield, Gagnon, & Jack, 2003), while temperature has contrasted effects on the onset and the end of the needle growing season (Gordo & Sanz, 2009; Olszyk, Wise, VanEss, Apple, & Tingey, 1998; Peñuelas, Filella, & Comas, 2002). Unlike Mediterranean semiarid vegetation that undergoes a long dry summer with reduced soil moisture, vegetation in New Mexico receives additional water during the Monsoon. Needle emergence of *P. edulis* in New Mexico occurs as early as May and can be postponed under heat/drought conditions until mid‐July, generally concomitant with the arrival of monsoonal moisture (Adams et al., 2015; Grossiord et al., 2016). We found that the total needle length (*l*) (end of the elongation period ~ October; Adams et al., 2015) was best explained by models including premonsoon and monsoon precipitation as predictors, and both had a similar weight in the models. Including a lag in the predictors did not increase the predictive power of the model. We suggest that winter rainfall supplies water for early spring photosynthesis and increases in carbon stocks, until soil moisture is depleted (Dickman, McDowell, Sevanto, Pangle, & Pockman, 2015). Early in the summer, monsoonal moisture increases turgor in the buds for leaf emergence and sustained expansion (Boyer, 1970; Korner, 2003; Palacio, Hoch, Sala, Korner, & Millard, 2014; Würth, Peláez‐Riedl, Wright, & Korner, 2005). This sensitivity to a bimodal precipitation period should be accounted for, when designing future irrigation experiment, and more broadly when inferring climate change influence on monsoonal vegetation.

Interestingly, our results showed that needles on droughted trees could reach the same length as on irrigated trees despite receiving at least 50% less precipitation. The drier the soil, the more responsive needle length was to interannual variability in water supply (i.e., βppt‐irrigated < βppt‐ambient < βppt‐droughted), emphasizing that the wetter treatment buffered interannual variability of needle length. Although droughted trees experienced lower predawn leaf water potential than the ambient treatment during the growing season (Pangle et al., 2015), they still produced needles of similar length. This suggests that factors other than predawn leaf water potential and turgor during the growing season are influencing *l*. Studies have suggested that the sizes of certain components of branch growth in current year depend on previous year precipitation (Clements, 1970; Löf & Welander, 2000). Nonetheless, we report similar *l* for all treatments in year 2012, with the ambient treatment having slightly lower *l* than the droughted one. We suggest that this could be the result of phenological phasing. Newly grown leaf area in a piñon branch is the result of successive phenophases (budburst, needle emergence and needle elongation)(Adams et al., 2015). These phases can reach various degrees of completion, giving the possibility to adjust the growth of new leaf area during the growing season. For example, droughted piñons can have a lower percentage of needle emergence during drought (Adams et al., 2015).

We analyzed the variation in yearly addition of *A*_S_:*A*_L_ (denoted SA:LA) and SA:LA/*d* and their responses to drought. Opposite to our hypothesis, irrigated trees maintained a significantly higher yearly SA:LA and SA:LA/*d* than other treatments. This result contrasts with previous studies on Mediterranean *Quercus ilex* that reported a significant increase of apical SA:LA, lasting up to 5 years after the experiment onset (Limousin et al., 2012; Martin‐StPaul et al., 2013). These contradictory results might stem from different properties of the primary xylem of the apical SA:LA (apical values were not considered in the present study), from the specificity of *P. edulis* and from the climate of New Mexico (monsoonal vs. Mediterranean). The importance of pre‐monsoon precipitation (i.e., mainly winter precipitation as snow fall) in explaining the interannual response in SA:LA and SA:LA/*d* is not surprising as snow pack melting provides soil moisture for photosynthesis, cell division, and shoot growth in early spring (Adams et al., 2015; Hallman & Arnott, 2015). The difficulty of selecting the best climate predictor between monsoon precipitation and the number of high VPD days (>4.5 kPa) during the monsoon is common because of the high coupling between these two climate variables. Unlike needle length, both functional ratios exhibited similar interannual variability across treatments (Table S7b,c) and their responses were robust to changes in precipitation and VPD, regardless of the treatment water status. Moreover, when dividing interannual variations of SA:LA and SA:LA/*d* by their means, differences disappear, emphasizing that the interannual response is simply linear (except for year 2011, see below). In addition, the yearly ratios exhibit no lag/memory, that is, influence of previous years, and primarily reflect dryness condition of the current year. It also suggests a tight balance between structural components—sapwood, leaf area, shoot length—might be sufficient in this isohydric species to cope with atmospheric or soil stress, similarly to recent results for isohydric grasslands (Konings, William, & Gentine, 2017).

### 4.2 Implications for leaf water potential

The annual changes in SA:LA should be differentiated from the long‐term adaptation expressed by *A*_S_:*A*_L_ and also differentiated from the stand‐level response (Martin‐StPaul et al., 2013). Our results suggest that multiple consecutive years of drought may create successive annual developments of evaporative structure with low SA:LA, thus cumulatively lowering *A*_S_:*A*_L_ of the piñon. This is in contradiction with the absence of difference found in piñons under different water/heat treatments (Grossiord et al., 2017) and with other intraspecies studies of conifers, that found a correlation between dry average conditions and higher *A*_S_:*A*_L_ (Callaway et al., 1994; DeLucia et al., 2000; Martínez‐Vilalta et al., 2009; Mencuccini & Bonosi, 2001; Whitehead et al., 1984). We see four reasons for these discrepancies. First, it is possible that higher values of *A*_S_:*A*_L_ reported for drier conditions—compared to values for wetter conditions—result from the acclimation achieved on time scales longer than the duration of our experiment (i.e., decadal or longer) (Martin‐StPaul et al., 2013). Second, it could be possible that a substantial proportion of the measured sapwood (*A*_S_) in branches may not be conducting, especially in the drought experiments, which would increase *A*_S_:*A*_L_ and diverge from the actual hydraulic allometry. This bias in *A*_S_ could explain the higher values of *A*_S_:*A*_L_ (~4.5 cm^2^/m^2^) compared to SA:LA in our study (~1.5 cm^2^/m^2^) (Grossiord et al., 2017; Hudson, 2016). Third, studies typically standardize measurements of *A*_S_:*A*_L_ by cutting branches at a fixed distance from the tip (e.g., 20 cm; Grossiord et al., 2017). Under experimental manipulation, the annual elongation of the shoot is expected to be reduced with drier conditions (Adams et al., 2015), likely resulting in measurement of *A*_S_:*A*_L_ that includes more years of sapwood in drier treatments, possibly introducing a dilution of experimental signal in the ratio. And fourth, drought‐stressed trees might develop low SA:LA during multiple years and still be able to maintain a cumulative *A*_S_:*A*_L_ that is higher than average. Indeed, cumulative *A*_S_:*A*_L_ also integrates the active regulation of leaf area (*A*_L_). Under dry conditions, a reduction in *A*_L_ can occur through leaf abscission even in evergreen species (Manzoni et al., 2015; Maseda & Fernandez, 2006; Munné‐Bosch & Alegre, 2004; Vico et al., 2014) and lead to an increase of *A*_S_:*A*_L_. However, in our experiment, this does not seem to be the case because branches on droughted trees maintained more years of needles (5.9 years, *SD* = 0.99) than branches on irrigated trees (5.4 years, *SD* = 0.81); Kolmogorov–Smirnov test, *p *=* *.21).

Under drought conditions, with no annual lag, pinions build new evaporative structure of reduced SA:LA and SA:LA/*d*. If acclimation might occur after few years (Martin‐StPaul et al., 2013), temporarily, low SA:LA contributes to a decrease in the leaf‐specific conductivity (*k*_L_) and therefore an increase in the water potential gradient for the same water transport (*E*, Equation (1)). In addition, when including the length of the new evaporative element within the framework of Equation (1), the linear resistivity of the branches increases which results in a bigger drop of water potential for the same level of evaporation. We conclude that the annual adjustments in the evaporative structure do not support a reduction in the water potential drop under soil dryness. Rather, trees try to increase the overall maximum sap and transpiration rates as SA:LA/*d* decreases across treatments.

We suggest that the relatively isohydric piñons do not need evaporative structure reduction to mitigate the drop of water potential under droughts (see below). When drought stress increases, piñons can still actively close stomata to reduce evaporation, preventing drop of leaf water potential regardless of *A*_*L*_ (Limousin et al., 2013). Stomatal closure, however, comes at the expense of lowering carbon assimilation (Limousin et al., 2013), which impacts piñons carbon status (Dickman et al., 2015) and further development of both LA and SA (Palacio et al., 2014).

### 4.3 Carbon allocation

Reducing yearly increments in SA:LA during drought emphasizes that, in relative terms, more carbon is being allocated toward leaves than xylem. Rather than creating a safer hydraulic structure supporting the regulation of leaf water potential by reducing leaf area, piñons tend to maintain photosynthesis by decreasing LA less. This response allows for more carbon gain outside of the growing season, when water is available and stomata are opened (Wright et al., 2004). The importance of LA for carbon gain and thus resilience and growth has been identified in other conifer species. It was shown that reduced LA is associated with lower stem and shoot growth (Albaugh, Allen, Dougherty, & Kress, 1998; O'Neil, 1962; Vose & Allen, 1988) and with a shift of carbon allocation toward storage (Wiley, Huepenbecker, Casper, & Helliker, 2013). Elevated carbohydrate production also increases tree defenses against biotic agents (McDowell et al., 2011), while more carbon being available for xylem growth may strengthen new xylem tracheids (Eilmann, Zweifel, Buchmann, Graf Pannatier, & Rigling, 2011; Martín‐Benito, Beeckman, & Cañellas, 2012) and therefore reduce vulnerability to cavitation (Bouche et al., 2014). Eventually, reduced allocation of carbon to xylem compared to leaves might also be the result of a strategy prioritizing the building of larger carbohydrate pools over xylem growth, saving resources for future growth (von Arx et al., 2017), or the result of an allocation strategy toward root development, in order to reach deeper soil layer and access more water (Gálvez et al., 2011). Overall, a reduced xylem growth together with a relative large photosynthetic surface area seems to have numerous advantages for pine development and maintenance under drought and does not necessarily imply an increase in drought stress similar to other pine species (Jacquet, Bosc, O'Grady, & Jactel, 2014).

Lower SA:LA in droughted trees might increase the carbon used for respiration. In *Pinus strobus*, mean respiration of foliage during the growing season is about double that of the sapwood (Vose & Ryan, 2002). Accordingly, droughted piñons in our experiment could have reached almost neutral leaf level carbon balance during summer days, with daily respiration matching daily assimilation (Limousin, Yepez, McDowell, & Pockman, 2015). This would suggest that droughted foliage may not have produced extra carbohydrates for woody biomass growth and/or maintenance.

### 4.4 Extreme drought and nonlinear responses

Simultaneous occurrence of extreme soil drought (low precipitation) and atmospheric drought (high VPD) in 2011 led to an extreme response in evaporative structure in trees from the ambient and droughted treatments. Under these conditions, piñons developed the shortest needles, a reduced leaf area, and the lowest yearly SA:LA and SA:LA/*d*. In semiarid regions, decoupling the impact of VPD from precipitation on evaporative structure can be difficult because high VPD and low precipitation are often strongly correlated (Novick et al., 2016). The strong correlation between precipitation and VPD during the monsoon does not allow us to statistically reject the effect of VPD on needle length and functional ratios. However, comparison of needle length, SA:LA, and SA:LA/*d* in three specific years (2009, 2011, 2013) supports the implication of VPD in the extreme response of evaporative structure. Ambient trees in 2009 had the same seasonal water input as irrigated trees in 2011 (~25% below the experimental mean, Figure 1a–c), and despite the extreme VPD in 2011, in both cases trees had similar needle length and yearly incremental SA:LA (Figures 3a and 6b). However, ambient trees in 2011 had similar seasonal water inputs as droughted trees in 2013 (~50% below experimental mean, Figure 1a–c), but needle length and SA:LA values in 2011 were half those in 2013 (Figures 3a and 6b). Mean monsoonal VPD in 2011 was 9% higher than the experiment average, while in 2013 it was 14% lower. These singular observations confirm that when atmospheric drought is combined with soil drought, foliar development is limited (Weiss, Betancourt, & Overpeck, 2012), and suggest that the evaporative structure response may become highly nonlinear. While little is known about the physiological disruption leading to these changes in evaporative structure, it is clear that climate extremes can significantly alter the annual functional ratios and challenge our ability to model the responses of structure and function in piñon pine.

## CONFLICT OF INTEREST

None declared.

## AUTHOR CONTRIBUTIONS

PG, MG, DMB, KG, and LAH contributed to the hypothesis conception and the design of the research. MG, RH, and RM conducted fieldwork. WP and NMD operated the experimental site and supported data collection. MG, RH, and GvA completed lab measurements. MG, DMB, GvA, and PG performed data analysis and produced interpretations. MG wrote, revised, and finalized the manuscript under the editing supervision of PG, with critical contributions by all authors. All gave final approval.

## DATA ACCESSIBILITY

Available data deposited in the Dryad Digital repository doi:10.5061/dryad.mr5p1.
