# Supplementary material for: Interannual variations in needle and sapwood traits of Pinus edulis branches under an experimental drought
Source: Ecol Evol. 2018 Jan 5;8(3):1655–72. doi: 10.1002/ece3.3743 (PMC5792598; doi:10.1002/ece3.3743)
Supplement: Supplementary file 1 [file ECE3-8-1655-s001.docx]

**SUPPORTING INFORMATION***Title: Interannual variations in needle and sapwood traits of Pinus edulis branches under an experimental drought*


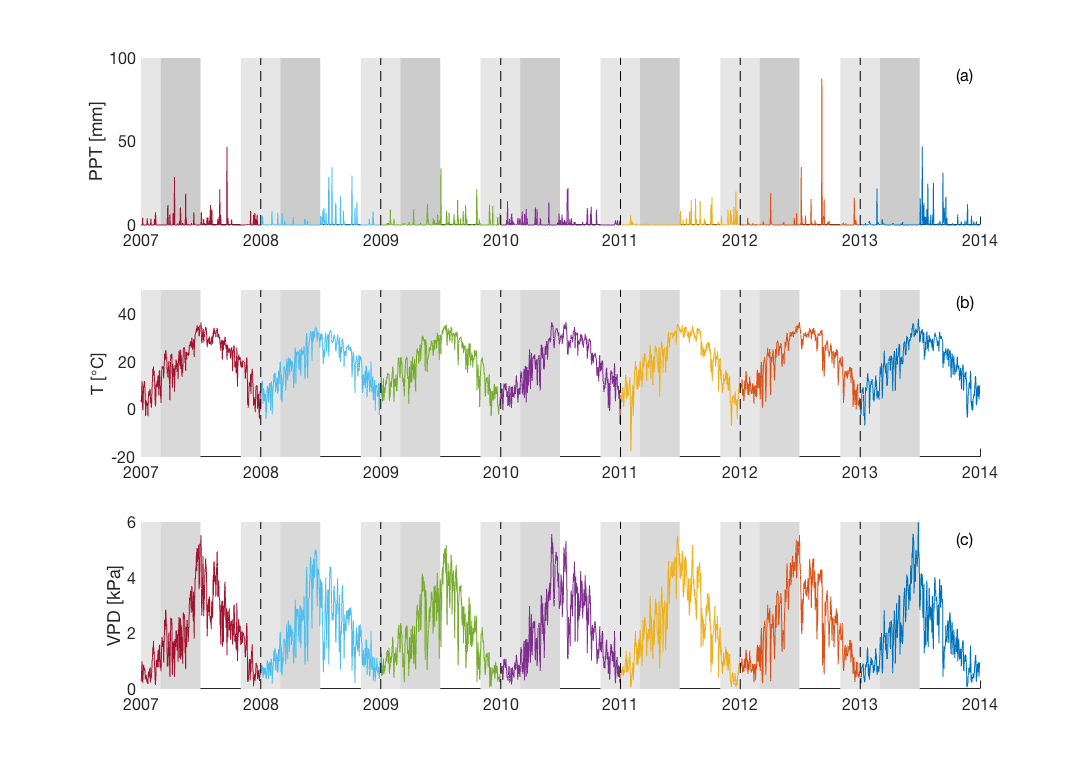


Fig. S1: Climatic data measured at Los Pinos Mountains of the Sevilleta National Wildlife Refuge, New Mexico, USA. (a) daily precipitation at the rain gage, (b) Temperature recorded at a 15 min time step, (c) maximum daily VPD derived from temperature and relative humidity. Light grey shading indicates winter period (Nov 1^st^ – Feb. 28^th^), darker grey indicates dry season period (March 1^st^ – June 30^th^), together, grey shadings highlight the period defined as pre-monsoon (Nov. 1^st^ – June 30^th^). Absence of shading corresponds to monsoon period.


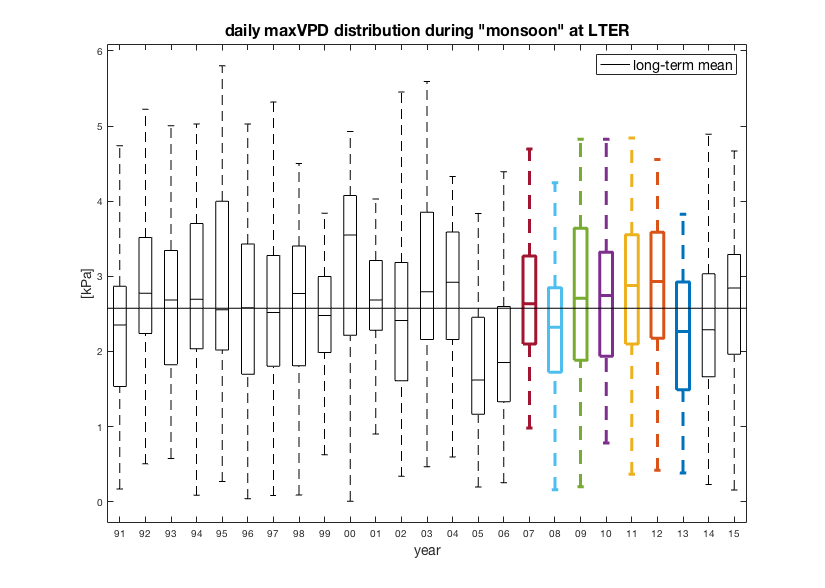


Fig. S2: Long-term distributions of maximum daily VPD during the dry season and monsoon, recorded at LTER, Cerro Montosa #42, New Mexico, USA (http://sev.lternet.edu - 25 years, 1991-2015). Colored box plots refer to years of the experiment. Horizontal line is the long-term [1991-2015] (*μ*=2.57, *σ*=0.3).


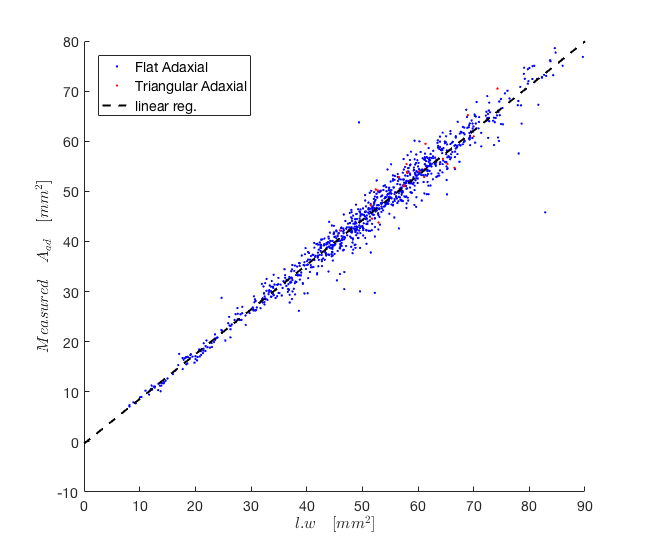


Fig. S3: Linear regression used to model flat Adaxial Area (*A_ad_*) in [mm^2^] based on length (*l*, mm) and width (*w*, mm) product (N=971, needles of *Pinus edulis*). *Measured* *A_ad_* was obtained from scanned flat needles (jpg, 1200 dpi) and Adobe Photoshop. Needles adaxial face was flat for most needles (blue), and sometime with triangular shape (red). Regression was *Measured* *A_ad_* *= -0.354 + 0.892*·*w*·*l*. Only flat adaxial faces were used for regression (*r*^2^=0.966).

Fig. S4: Raw temporal series of Mean Ring Width (MRW) for the largest section of each branch. Knowing the innermost ring of each section (due to the location of the cut on a specific yearly branch segment), and knowing the outermost ring as well (2013), we could visually assign the rings to specific calendar years. 2011 (extreme drought) appears to be the only missing ring in our dataset, confirming the ease of ring identification.

Fig. S5: Zoom of Fig. 5.

Table S1: Design of samples and measurements

Table S2: Measurements and tree details

Table S3: List and definition of predictors used in Linear Mixed Effect Models (LMEM).

Table S4: List of models tested in the structured approach, in order to identify best models for *l*, and . We first compared different linear models together using ANOVA. We then investigated the increase of fit made with LMEM and concluded on the best models (in light blue).

Table S5: Pearson’s correlation coefficients and p-values between cumulative precipitation and number of days with VPD > 4.5 kPa. Precipitation being pre-monsoon (Nov. 1^st^ to June 30^th^) and monsoon (July 1^st^ to Oct. 31^st^). VPD days counted during the dry season (MAMJ), during the monsoon (JASO). Time series was taken from 2007 to 2013 at the experimental site. Between parentheses is the p-value.

Table S6: Linear regressions of *l*, , measured from 29 branches on the period 2007-2013. Equations are of the form *Y = int. + α-treatment*·*X*. Needle length *l* is in mm, in cm^2^·m^-2^, in m^2^·m^-3^, with *n* the sample size, r2-adjusted: coefficient of determination adjusted for degree of freedom. F-statistic: overall F-test for the regression. P-value: significance of overall F-test. *: *p-value*<0.05.

Table S7: The following tables summarize the bootstrap results obtained from Linear Mixed Effect Linear Modeling of needle length (a.), (b.) and (c.) using two climate variables as predictors. The horizontal black lines indicate one normalized climate predictor, grey left panels indicates the second normalized predictor. Each predictor was used as fixed and random effect; the grouping variable was the treatment (e.g. *Y = 1 + ppt + vpd + (1 + ppt + vpd | treatment)*). See Annex S3 for reading guidance.

Table S8: Linear regressions of on as presented on Fig. 5 and Fig. S5. Intercept was forced to zero and slope were compared between treatments.

is obtained by combining the data from branch structure and needle structure. Between the wood section cut and the branch’s tip, the total number of needles created in year *y* is multiplied by the mean leaf area (2·A_ad_) of needles created in year y on this same branch, totalizing as . In this calculation it is assumed that there is no significant difference between mean leaf area obtained from needles on the primary axis of the branch and from needles taken on secondary axis. It is also assumed that the abaxial face has no curvature, and therefore the needle area is the double of the adaxial area.

measurements were limited by . When needles of certain years were absent on the branch we used for sapwood area, we retrieved missing needle areas by using needle areas from other branches of the same tree (3 branches per tree were collected). Retrieval was a linear interpolation using the Matlab function “scatteredInterpolant”. If the other branches were lacking the same needles area, no interpolation was made. These interpolations were only used for and allowed to derive 6 additional, totalizing 163 values.

From ***Material and methods*** section, we presented as the mean branch for year *y*, obtained by averaging the yearly of the different wood sections of the branch. From observations, it appeared that for a fixed year y, (first year of growth, Γ=y) was significantly higher than (same year but measured from older cuts, Γ < y). Therefore, when averaging we discarded the values corresponding to 1^st^ year of growth, . In other words, we make the assumption that the measured at the very tip of the branch is not representative of variations in the rest of the branch and should not be included in our interannual study.

The branch structure was reported for the same branches that were used for the measurement of sapwood area. In primary and secondary axis, we counted (i) all needles in each growth segment – when present; (ii) all needles’ scars – when needles had been dropped; (iii) all dots from which needle never emerged. Summing (i) and (ii), we then computed precisely the total leaf area built each year between the tip of the branch and the location of the wood slices precisely the total leaf area built each year between the tip of the branch and the location of the wood slices.

Annex S1: Deriving

is the annual ratio of newly built sapwood by newly built leaf area, divided by the distance to the new tip. It is derived by combining the data from the evaporative anatomy (, in m^2^·m^-2^) and the distance to the tip (, in m). On the same branches, was measured between the wood section where was measured and the tip of the branch, as it was in year *y*. Therefore, except for year 2013 where distance to tip was readily measurable on the freshly cut branches, previous years tip position were retrieved by subtracting elongation segments to 2013’s tip (Fig. 2).

From ***Material and methods*** section, we presented as the mean branch for year *y*, obtained by averaging the yearly of the different wood sections of the branch. Similarly to when averaging we discarded the values corresponding to 1^st^ year of growth, so that . In other words, we make the assumption that the measured at the very tip of the branch is not representative of variations in the rest of the branch and should not be included in our interannual study.

Annex S2: Deriving

Annex S3: A bootstrap approach was used to strengthen results obtained from the Linear Mixed Effect Model (LMEM). The underlying assumption of LMEM is the normality of residuals and coefficients. Bootstrap is a non-parametric test that allowed confirming the significance of predictors coefficient obtained with LMEM. It also allowed testing the differences of response (coefficients) across treatments, regardless of the normality assumption.

We first standardized the predictor variables, VPD and precipitation, over the different periods (yearly value minus experimental mean divided by standard deviation). We pull randomly with replacement 1,000 datasets from the original dataset. For each dataset we ran LMEM using precipitation and VPD as fixed and random effect. We obtained from each run 3 coefficients for each treatment (intercept, βvpd and βppt). From the 1,000 runs we obtain for each treatment a distribution of (intercept, βvpd-treatment, βppt-treatment). These distributions were used for:

- testing whether a predictor coefficient (e.g. βvpd-irrigated) was significantly different from zero.
- testing for each treatment if the influence of VPD and precipitation was significantly different (e.g. |βvpd-irrigated| > |βppt-irrigated|).
- testing for if VPD effect in one treatment was significantly different from VPD effect in another treatment (e.g. |βvpd-irrigated| > |βvpd-droughted|).
- testing for if precipitation effect in one treatment was significantly different from precipitation effect in another treatment (e.g. |βppt-irrigated| > |βppt-droughted|).

The 1,000 runs also allow deriving a mean performance index for AIC, BIC and Logelikelihood.

***

Size of the population for Y = “needle length” is 173 data points (58 irrigated, 61 ambient, 54 droughted, measured from 29 branches between 2007 and 2013).

Size of the population for Y = “SA:LA” and SA:LA/d is 163 data points (48 irrigated, 58 ambient, 57 droughted, measured from 29 branches between 2007 and 2013).

***

Results for each bootstrap simulation are summarized in three rectangles.

- *Coefficients LMEM*: for each predictor in each treatment (βvpd and βppt-treatment), we tested if β was significantly different from zero (noted ≠0), and report the sign of the median of the β distribution obtain from 1000 runs.
- *Comparison of coefficients*: the left side coefficient is compared with the right side coefficient from the distribution of 1000 models. “~ “ means not significantly different, “< or >” means significantly different at the 5% level.
- *Bottom rectangle*: mean performance results when averaging over the 1000 runs.

*Example of bootstrap reporting for Y=needle length modeled with pre-monsoon precipitation and monsoon VPD (number of days > 4.5kPa during JASO). Green reporting of performance index means higher performance than black reporting.*

Performance of the 1000 LMEM is measured with *AIC, BIC* and *LogLikelihood* statistics. We reported the mean over the 1000 runs : *AIC_mean, BIC_mean, LogLik_mean.* Below are the densities of these statistics.


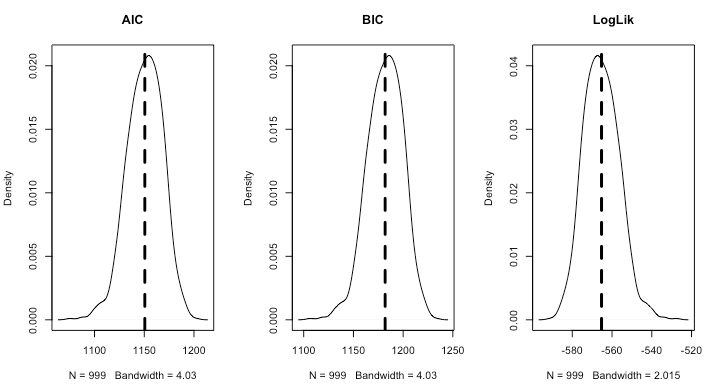


For precipitation predictor, each Linear Mixed Effect Model (LMEM) generates one coefficient per treatment for each predictor (*βppt-treatment* or *βvpd-treatment*). For each coefficients, we obtain their distribution from the 1000 runs. If 0 is not between the 0.025^th^ percentile and the 0.975^th^ percentile, we reject the null hypothesis at the 5% level (H0: β =0) and report “≠0” with the sign of the coefficient (“+” or “-“) in the “coefficient LMEM” section. Otherwise, we report “≈0”.


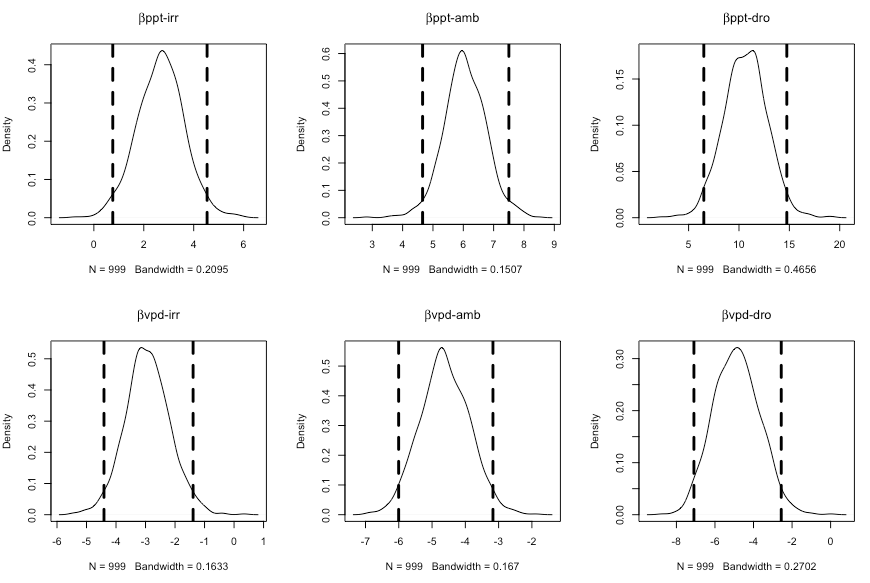


We test if two *βppt-treatment* are significantly different at the 5% level. If 0 is not between the 0.025^th^ percentile and the 0.975^th^ percentile, then we can reject the null hypothesis at the 5% level (e.g. H0: *βppt-irrigated* - *βvpd-droughted = 0*) and report “>” in the “comparison of coefficients” section. Otherwise, we report “~”. Same approach and reporting is used to test coefficient s for precipitation obtain for each treatment (*βppt-treatment*) are significantly different from the coefficient of VPD (*βvpd-treatment*) at the 5% level.


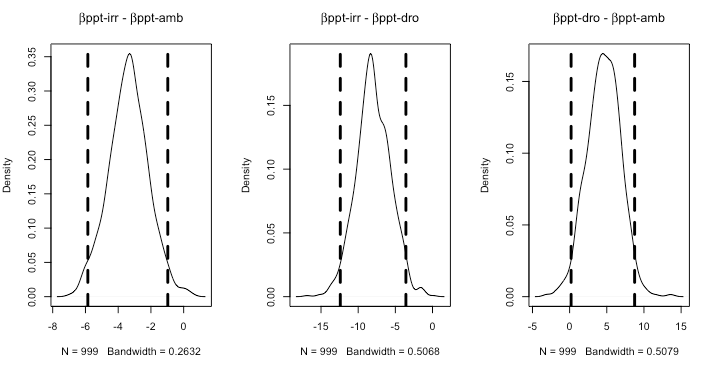


Annex S4: Example of R code used to run a bootstrap on Linear Mixed Effect Model (LMEM) with different combination of predictors.

- *needle_stz* : the matrix where each line contains a needle length (*length*) average and associated VPD (*vpd*) and precipitation (*ppt*), standardized by there mean and standard deviation. 168 lines in total.
- *needle_stz_samp* : is a random selection of lines taken with replacement in *needle_stz*.
- *coef_lmm* : stores the coefficients obtained for *intercept*, *vpd* and *ppt* in each treatment when running LMEM with *needle_stz_samp* dataset (1000 runs here).
- *stat_lmm* : a matrix that stores for each LMEM the AIC, BIC and LogLik (1000 runs here).

**# Bootstrap ---- NEEDLE LENGTH --------------------------**

coef_lmm=array(NA, dim=c(3,3,1000))

coef_lmm_error=matrix(nrow=1000,ncol=1)

stat_lmm=matrix(nrow=1000,ncol=3)

err<-"Error"

for (i in 1:1000){

needle_stz_samp=needle_stz[sample(nrow(needle_stz),size=nrow(needle_stz),replace=TRUE),]

a<-try(lme(length~1+ppt + vpd, random= ~1+ppt + vpd |treatment, data=needle1_stz_samp,control = lmeControl(opt='optim')))

a<-str_sub(a,1,5)

if (identical(a,err) ==TRUE)

{coef_lmm1_error[i,]=i }

else {

coef_lmm1_no_error[i,]=i

lmm_stz_samp<-lme(length~1+ppt + vpd + vpd, random= ~1+ppt|treatment, data=needle_stz_samp,control = lmeControl(opt='optim'))

coef_lmm1[,,i]=as.matrix(coef(lmm_stz_samp))

stat_lmm1[i,1]=AIC(lmm_stz_samp)

stat_lmm1[i,2]=BIC(lmm_stz_samp)

stat_lmm1[i,3]=lmm_stz_samp$logLik}

}

message("Number of samples that did not converged ", length(coef_lmm1_error[!is.na(coef_lmm1_error[,1]),]))

When 1000 samples were run, we obtained a distribution for each coefficient of each predictor (β1, β2) and for each treatment. We could test (i) if a coefficient was significantly different from zero (5% level) by confirming zero was smaller than the 0.025 percentile or larger than the 0.975 percentile; (ii) with the same method, we could test if the coefficient obtained from two different treatment were significantly different (5 % level). We could also get the mean AIC, BIC and log-likelihood obtain from the 1000 runs.
